# Applications of Isothermal Titration Calorimetry in Studying Biomimetic Nanocarriers

**DOI:** 10.3390/biom15101349

**Published:** 2025-09-23

**Authors:** Martin Guerrero, Colby Braden, Yuping Bao

**Affiliations:** Department of Chemical and Biological Engineering, The University of Alabama, Tuscaloosa, AL 35487, USA; mguerrero1@crimson.ua.edu (M.G.); cwbraden@crimson.ua.edu (C.B.)

**Keywords:** biomimetic, nanocarriers, ITC, liposome, EVs

## Abstract

Biomimetic nanocarriers, particularly membrane-based systems, have emerged as promising platforms for drug delivery. A thorough understanding of the molecular interactions that govern their assembly, stability, and cargo-loading efficiency is essential for optimizing their design and performance. Equally important are their interactions with biological components such as proteins, lipids, nucleotides, and cells, which significantly influence delivery efficacy. Among various techniques for characterizing these nanocarriers, isothermal titration calorimetry (ITC) has proven to be an invaluable tool to study their molecular interactions. ITC enables direct quantification of key thermodynamic parameters, such as binding affinity, stoichiometry, enthalpy, and entropy changes, without the need for molecular labeling or immobilization. This review highlights the application of ITC in the study of biomimetic nanocarriers, focusing on solid lipid nanoparticles, liposomes, extracellular vesicles, cell-derived vesicles and live cells. For each type of nanocarrier, the ITC applications in specific areas and the resulting information are discussed. For example, ITC was used to characterize drug interaction and protein adsorption for solid nanoparticles. In contrast, many aspects of liposomes were explored by ITC, including membrane solubilization and stabilization, peptide interactions, and macromolecule and protein adsorption. Overall, this review aims to provide a conceptual and practical framework for employing ITC in the investigation of biomimetic nanocarrier systems, facilitating their rational design and improved therapeutic performance. Furthermore, the discussion encourages further development of strategies to increase the application in cell-derived vesicles and live cells.

## 1. Introduction

Biomimetic membrane-based nanocarriers represent a promising approach in nanomedicine for drug delivery [1,2,3]. Here, the term ‘biomimetic’ refers to nanosized structures derived from or coated with natural cell membranes and is used interchangeably with the concept of membrane nanocarriers. Compared to conventional drug delivery systems, these nanocarriers offer enhanced biocompatibility [4], improved immune evasion [5], and capability for specific targeting [5]. Depending on the origin of membrane lipids and surface engineering strategies, nanocarriers with desirable properties can be achieved, such as prolonged circulation times and improved biodistribution [6]. By bridging the gap between engineered platforms and natural systems, biomimetic nanocarriers serve as powerful tools for next-generation therapeutics and diagnostics. A deep understanding of the molecular interactions of biomimetic membrane-based nanocarriers is critical for optimizing their design, targeting efficiency, and therapeutic performance. These interactions further dictate their engagement with biological targets, such as cells, proteins, and membranes, and subsequently influence their biodistribution and therapeutic efficacy. Numerous techniques have been employed to study biomimetic nanocarriers, including stability, drug encapsulation efficiency, and interactions with biological components. For example, surface plasmon resonance, an optical technique, is widely used to measure the binding kinetics and affinity of molecules on nanocarrier surfaces; but, it requires immobilization of one interaction partner [7,8]. Quartz crystal microbalance can characterize interactions between nanocarriers and proteins, ligands, or cell membranes by detecting mass changes upon binding [9]. Fluorescence resonance energy transfer provides an effective way to study drug loading and release based on molecular proximity changes during binding events [10]. Microscale thermophoresis measures the movement of molecules in a temperature gradient, enabling sensitive detection of protein–nanocarrier interactions [11,12]. In addition, some separation-based methods were also explored to study binding affinity by quantifying the bound and unbound nanocarriers. For example, high-performance liquid chromatography with immobilized protein as stationary phases can be used to determine nanocarrier–protein binding affinities from retention times [13]. Capillary electrophoresis frontal analysis was used to quantify affinity and stoichiometry of nanocarrier–drug or nanocarrier–nucleic acid complexes [14]. Field-flow fractionation separates nanocarriers based on size and composition, allowing assessment of binding events and heterogeneity within nanocarrier populations [15]. Unfortunately, these techniques only generate certain information related to thermodynamic parameters of interactions. In contrast, isothermal titration calorimetry (ITC) offers unique advantages [16]. First, ITC does not require labeling of nanocarriers or binding molecules, thus avoiding potential artifacts introduced by tags or fluorophores. Second, ITC provides direct, real-time measurements of molecular interactions and yields a complete set of thermodynamic parameters, including binding affinity, enthalpy, entropy, and stoichiometry. Traditionally, ITC has been widely used in drug discovery to study the binding affinity between drug candidates and target proteins, aiding in the rational design and optimization of therapeutics [17,18]. Recently, the applications of ITC have expanded into diverse biological areas [19], with comprehensive reviews covering soft materials [20], pharmaceutical technology [21], enzymatic reactions [22,23], nanoparticles [24,25], peptide–lipid membrane interactions [26], and membrane proteins and lipids [27,28]. Despite the growing interest in biomimetic membrane-based nanocarriers, a review of ITC applications in this area is still lacking.

Here, we first outline the ITC principles and discuss the key parameters influencing the ITC results. Then, a detailed discussion of ITC applications in characterizing different types of biomimetic nanocarriers is provided. The various biomimetic nanocarriers will mainly be membrane lipid-based structures, focusing on solid lipid nanoparticles, liposomes, extracellular vehicles (EVs), and cell membranes. For each type of nanocarriers, the discussion is based on the available studies. For instance, ITC has been used to investigate protein adsorption on solid lipid nanoparticles, and the subsequent impact on targeting efficiency. In the case of liposomes, ITC studies have been explored in many aspects, including structural stability, cargo loading, interactions with various molecules. In contrast, only a limited number of ITC studies are available for EVs despite their great promise as drug delivery vehicles and diagnostic tools. Interestingly, it is also feasible to study ligand–cell surface receptor interactions, monitor metabolic activity for whole live cell systems using ITC. Through these discussions, this review aims to encourage further exploration of ITC for characterizing biomimetic systems, particularly EVs and live cells.

## 2. Isothermal Titration Calorimetry

### 2.1. Principle of ITC

**Figure 1:**
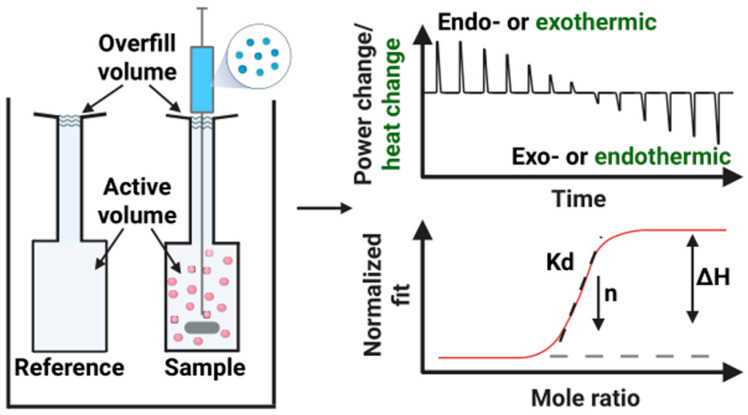
Illustration of the ITC working principle, representative thermograms, and the data fitting curve. The binding isotherms are generated by integrating the peak areas from each injection. These data are then fitted to appropriate models to determine Kd, ΔH, and *n*, while ΔS and ΔG are calculated using standard thermodynamic relationships. Sequential injections enable the construction of complete binding curves, allowing for accurate determination of binding affinity, stoichiometry, and cooperativity. The dash line indicates the baseline. Created with https://www.biorender.com/60ott6k.

ITC is a powerful analytical technique that measures heat changes during chemical or biochemical reactions, providing comprehensive thermodynamic information about the molecular interactions [16]. Key parameters obtained from ITC measurement include the dissociation constant (Kd), which reflects the strength of the interaction; enthalpy change (ΔH), representing the heat absorbed or released during the reaction; entropy change (ΔS), indicating the degree of disorder or randomness in the system; and stoichiometry (*n*), the ratio of interacting molecules [29]. Generation of a complete set of thermodynamic parameters is a key advantage of ITC technique, which allows quantifying molecular interaction in a single experiment without the need for labeling or immobilization. In a typical experiment, one reactant is incrementally titrated into a sample cell containing the other reactant under gentle stirring. A reference cell, filled with the same solvent for reaction, is used to correct for background heat changes. As each titration proceeds, the instrument continuously records the heat released or absorbed, producing a characteristic thermogram (or titration curve) as a function of the molar ratio of the reactants. The resulting data can be analyzed and fitted into various binding models (e.g., simple 1:1 interactions, sequential binding, or multiple-site interaction) to extract detailed thermodynamic parameters. Figure 1 illustrates the working principle of ITC, including representative raw data, fitted curves, and the associated binding information.

Here, the volume of the reaction/reference cells is determined by the instrument types. For example, the cells of the affinity ITC (TA instruments, waters) standard version can be up to 1 mL while the low-volume version only holds up to 190 µL. The thermograms obtained from ITC experiments represent raw data showing the heat released or absorbed during molecular interactions. These thermograms can be displayed as either the power change in the instrument or the heat change within the reaction cell where the instrument’s power change is inversely related to the heat change (Figure 1). As a result, a positive peak in the power signal indicates an endothermic process where the reaction adsorbs heat from the environment, corresponding to a positive enthalpy change in the reaction but a negative heat value. Conversely, a negative power peak suggests an exothermic process—where the reaction releases heat to the surroundings, corresponding to a negative enthalpy change in the reaction and a positive heat value. Therefore, careful interpretation is required when determining whether a reaction is endothermic or exothermic, depending on whether the thermogram presents instrument power or heat rate. ITC measures binding reaction heat indirectly by adjusting the electrical power needed to maintain the sample cell at a constant, pre-set temperature. During an exothermic reaction, heat is released, causing the temperature of the sample cell to rise. To compensate, the instrument reduces heater power, resulting in downward peaks. Conversely, endothermic reactions absorb heat, leading to a drop in the sample cell’s temperature. In this case, additional power is required to maintain the set temperature, producing upward peaks. The area under each peak corresponds to the heat change per injection. By integrating these values as a function of the titrant-to-sample molar ratio, one can construct the binding isotherm and extract key thermodynamic parameters. Therefore, the ΔH is the parameter that can be directly obtained from normalized firing of the ITC thermograms. The binding constant is derived from the slope of the fitting curve. The shape of the binding isotherm is determined by the critical parameter c, a unitless constant. This constant is a product of the binding constant, initial concentration of the molecules in the sample cell and the stoichiometry, *n*, of the binding reaction. ΔS and ΔG are calculated using standard thermodynamic relationships.

Additionally, the trend of the peaks provides further insights into binding and equilibrium processes of the reaction, where the height and area of the peaks on the thermogram are directly proportional to the heat released or absorbed during the binding reaction. Larger peaks indicate a stronger interaction (higher binding affinity) and a greater heat change. The integrated area under the peaks on the thermogram reflects the enthalpy of binding. As the reaction approaches equilibrium, the peak height gradually decreases due to the reduced binding rate resulting from saturation of the available binding sites. From the thermograms, only enthalpy information can be obtained. To obtain thermodynamic parameters, a fitting model needs to be selected to fit the raw data. Depending on the availability of binding sites and binding kinetics, proper models need to be selected, such as simple 1:1 binding model, multiple site binding, sequential binding, etc. The quantitative thermodynamic parameters allow evaluation of the strength of molecular interactions.

Beyond the binding affinity, the enthalpy and entropy contribution offer deeper understanding of the nature of the interactions. Weak interactions can be driven by either enthalpy or entropy, or a combination of both depending on the nature of the interacting molecules, temperature, and solvent [30]. Enthalpy-driven interactions (Δ*H* < 0 and Δ*S* < 0) typically tend to be strong and exothermic, leading to a more ordered, stable structure, that can result from hydrogen bonding and van der Waals forces [31,32]. On the other hand, entropy-driven interactions (Δ*H* > 0 and Δ*S* > 0) tend to be weaker, such as the hydrophobic effects, often leading to a more diffuse or less stable structure [31]. The interactions with Δ*H* < 0 and Δ*S* > 0 could correspond to electrostatic interactions [32]. Therefore, ITC technique is a highly useful tool to study the molecular interactions via non-covalent, weak interactions.

### 2.2. Important Parameters Affecting ITC Results

**Figure 2:**
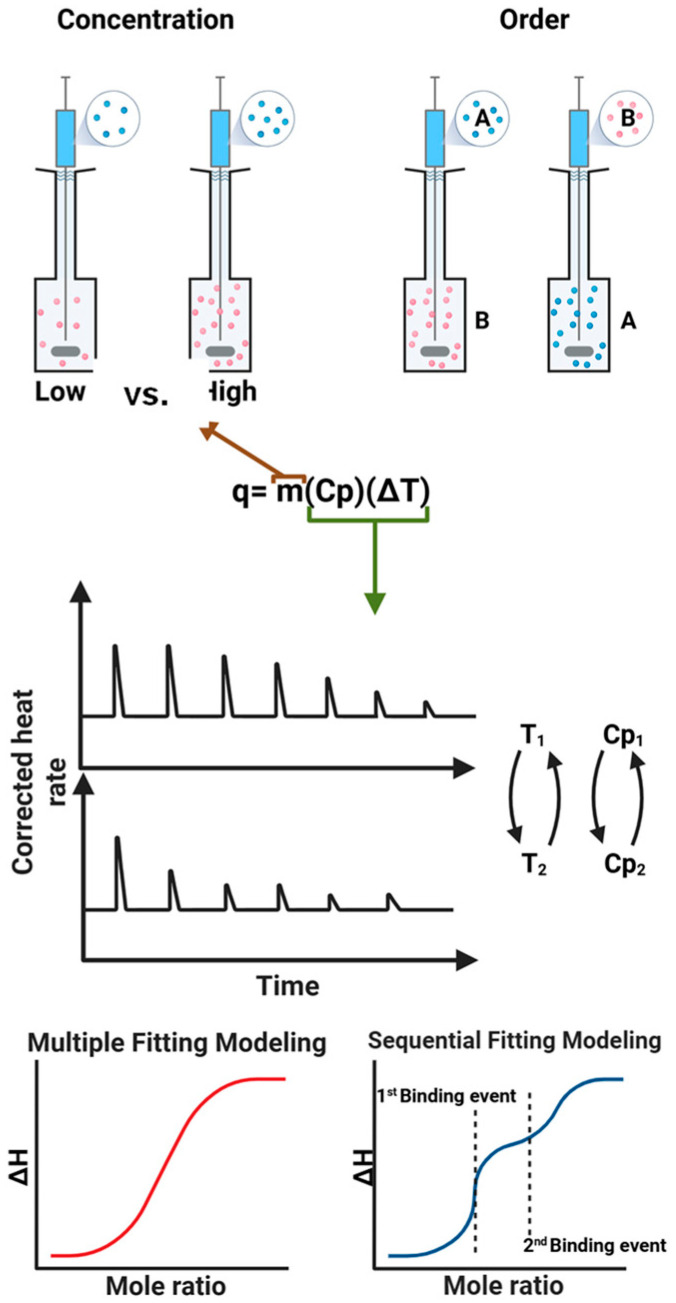
Schematic illustration of factors affecting the ITC experimental results. Representative thermograms highlight the influence of concentration and titration order. Baseline-corrected profiles reveal either larger peaks—indicating more binding events—that diminish with increasing saturation, or smaller peaks to peak plateau that reflect reaction completion. These data are typically fitted to a binding model (e.g., multiple-site or sequential binding model) to derive key thermodynamic parameters. Created in https://www.biorender.com/b1pmarp.

ITC technique measures the thermodynamics of molecular interactions by directly detecting heat changes during binding events; therefore, experimental designs ensuring precise measurements of heat exchange and proper calibration of the instrument are critical. To obtain reliable and interpretable data, several experimental parameters [33] must be carefully considered, as illustrated in Figure 2, such as solvent [34], temperature [35,36], concentrations of the reactants [33], and the selection of proper data fitting models [12,37].

ITC experiments are highly sensitive to temperature variations, and the heat capacity of solvents impacts the instrument’s ability to detect small heat changes. Minor fluctuations in temperature can introduce noise or artifacts into the data. Differences in heat capacity between the sample and the reference cell can lead to baseline drift or poor signal resolution [38]. In addition, the concentrations of both reactants need to be optimized for optimal heat generation. If the concentration of either reactant is too high, the binding sites can be saturated quickly, making it difficult to accurately determine binding parameters. On the other hand, too low a concentration of either reactant may result in weak signals, reducing the sensitivity of the measurement. The molar ratio of two reactants also needs to be appropriately set. For the best results, make the starting concentration as close as possible to the large-signal limits of the instrument. The number of injections (>10) is also important for better curve fitting [33], too large or too many injections may saturate the system quickly, preventing accurate model fitting. Optimal titration design (typically 20–30 injections) is necessary for good curve resolution.

Importantly, these parameters often correlate with one another. For example, temperature not only influences reaction kinetics but also affects solvent viscosity and heat capacity, which in turn impact the magnitude and shape of the heat signals detected. Similarly, beyond its effect on solubility, dilution heat can vary significantly between solvents, directly influencing the baseline, the amplitude of thermograms, and the sensitivity to reaction heats. Therefore, ITC experimental conditions must be optimized collectively to ensure accurate and reliable results.

In addition to experimental conditions, the binding site fitting model for ITC data analysis plays a crucial role in determining the thermodynamic parameters derived from an experiment [39]. If the number of binding sites of a molecule is known from prior knowledge, more stable and reproducible fitting can be readily obtained. On the other hand, if the stoichiometry needs to be estimated from data fitting, the proper choice of the model will be critical to obtain reasonable parameters. In such cases, the shape of the thermogram provides the initial guide with sigmoidal curves typically for single-site binding, and biphasic or plateaued responses for multiple binding events or allosteric effects. Single-site model can oversimplify complex interactions and ignoring multiple binding events can lead to incorrect thermodynamic interpretations. Multiple-site models account for different affinities or cooperative effects but require higher-quality data and more robust fitting routines [40]. The multiple site models can also lead to large fitting errors, but the resulting parameters (when reliable) better reflect biological reality. Therefore, choosing the appropriate binding site fitting model is essential for correctly interpreting ITC data. Inappropriate fitting choices can significantly distort the derived thermodynamic parameters, leading to incorrect biological conclusions [41]. A systematic study of uncertainty quantification in ITC data due to heat and concentration error suggested that proper experimental designs are critical to reduce experimental uncertainty by using more injections and by fixing stoichiometry at its known value [42]. Finally, periodic instrumentation validation with standards is important to minimize experimental errors [43]. Additionally, even if a model fits well statistically, it should also make biophysical sense. Therefore, ITC data are often further validated with orthogonal techniques (e.g., spectroscopy, mass spectrometry, structural biology) when possible. Under carefully designed experimental conditions, ITC measurements typically produce reliable Kd in the range of 100 µM down to 1 nM with a standard deviation (SD) around 5% [44]. Although the lower SD value indicate high confidence, the actual SD depends on the experimental setup and data quality, which can vary from 2–10%.

**Figure 3:**
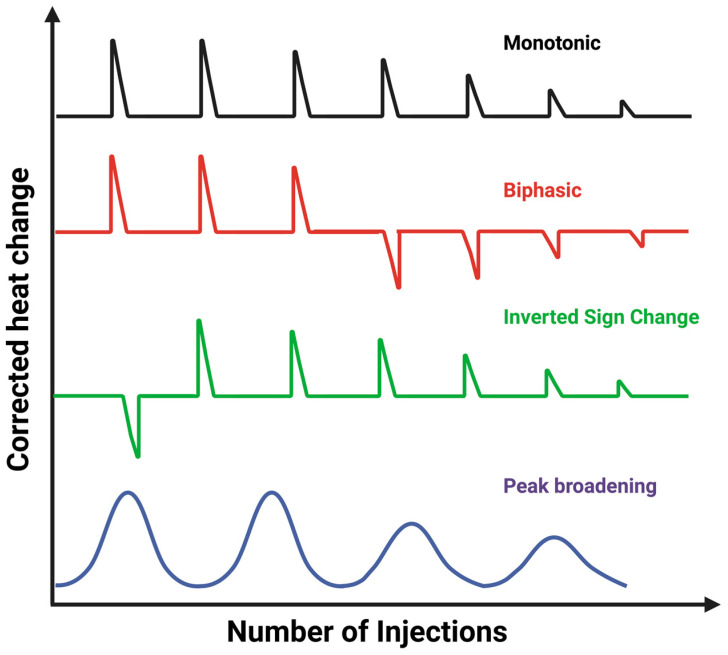
Representative ITC profiles examples of monophasic, biphasic, inverted sign-change, and peak-broadening thermograms. Created in https://www.biorender.com/ca9yy5j.

In a typical ITC experiment, the thermogram can be monophasic that fits well with a one-site binding model [45], or non-canonical profiles that deviate from this ideal behavior and reveal additional mechanistic complexity [46], as shown in Figure 3. One common example is the biphasic profile, in which two distinct binding phases appear during the same titration. This often arises when a macromolecule possesses more than one binding site or undergoes sequential binding events with different affinities. Another non-canonical case is the inverted sign-change profile, where the apparent enthalpy of binding shifts from exothermic to endothermic (or vice versa), typically reflecting conformational rearrangements, proton exchange, or other coupled processes that influence the net heat signal [47]. Peak broadening is another frequently encountered deviation, characterized by unusually wide or poorly resolved injection peaks. This effect can result from slow binding kinetics, aggregation, or incomplete equilibration between injections, all of which complicate data analysis. Recognizing such atypical thermograms is crucial because they signal that simple one-site models are insufficient. Instead, more advanced fitting strategies or complementary biophysical techniques may be required to extract reliable thermodynamic parameters and accurately interpret the underlying molecular mechanisms [45,46].

## 3. ITC Applications of Biomimetic Nanocarriers

**Figure 4:**
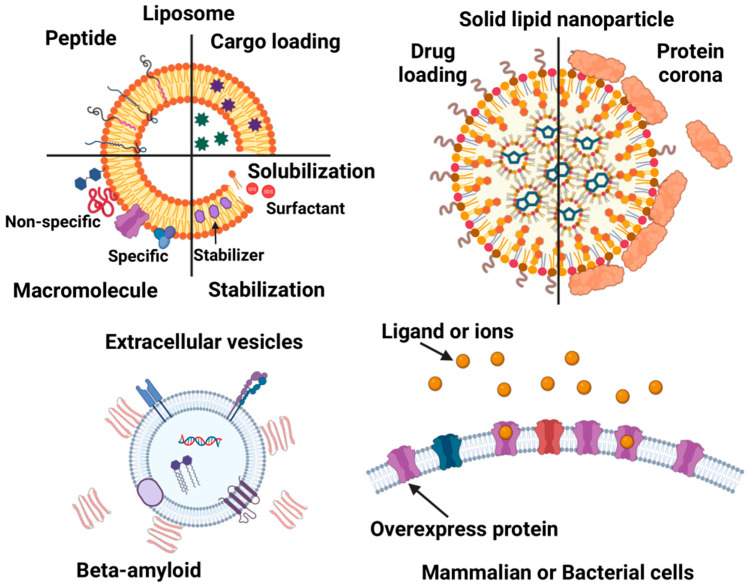
An overview of discussed biomimetic nanocarriers, including interactions of liposomes with peptides, polymers, surfactants, and drugs, solid lipid nanoparticles with surface protein adoption, protein corona formation and drug loading, extracellular vesicles and cell membrane–derived systems engage in processes such as ligand binding, molecular transport, and receptor-mediated interactions. Created in https://www.biorender.com/dkjpxo1.

The molecular interactions between biomimetic nanocarriers and biological systems critically modulate their circulation time, biodistribution, and ultimate fate, key factors for effective drug delivery. In the literature, these biomimetic nanocarriers are described using various terms such as lipid nanoparticles, liposomes, lipid vesicles, and membrane vesicles. For the purpose of this review, we adopt a specific nomenclature: lipid nanoparticles refer exclusively to solid lipid nanoparticles, while liposomes broadly encompass all membrane vesicles composed of synthetic and/or natural lipids. In contrast, EVs derived from cells, along with membrane vesicles generated via cell membrane reconstruction, are grouped together with live cell analyses. Our focus here is on ITC studies investigating the molecular interactions of solid lipid nanoparticles, liposomes, EVs, and membrane vesicles/live cells. Figure 4 provides an overview summarizing the discussion points for each nanocarrier type based on the available research.

### 3.1. ITC Applications of Solid Lipid Nanoparticles

Solid lipid nanoparticles represent a highly promising class of drug delivery nanocarriers due to their biocompatibility, biodegradability, and structural similarity to natural lipids [48]. These characteristics are particularly advantageous for the delivery of RNA-based therapeutics. The development of COVID-19 mRNA vaccines stands as a landmark example of the successful application of solid lipid nanoparticles in clinical settings [49,50]. Typically, solid lipid nanoparticles are composed of a solid lipid core that facilitates drug encapsulation, a surfactant layer that ensures colloidal stability and emulsification, and, in some cases, additional surface modifications to enhance biocompatibility or enable targeted delivery. Importantly, they are designed to remain stable at physiological and moderately elevated temperatures (25–45 °C), since higher temperatures can trigger lipid phase transitions and compromise nanoparticle stability. A detailed understanding of the molecular interactions between solid lipid nanoparticles, therapeutic agents, and biological environments is essential for optimizing nanoparticle design and maximizing therapeutic efficacy.

During the development of solid lipid nanoparticles, ITC has proven to be a valuable tool for elucidating the thermodynamics of molecular interactions, providing key insights into structural stability, drug encapsulation efficiency, and targeting capabilities. For instance, Huang et al. effectively investigated the interactions between weakly hydrophobic drugs and model lipids relevant to solid lipid nanoparticles using ITC [51]. In this study, benzoic acid and salicylic acid, two structurally similar weakly hydrophobic drugs differing only by a single hydroxyl group, were selected as model compounds, while stearic acid served as the representative lipid. The ITC thermograms revealed that both drug-lipid interactions proceeded through two sequential phases around a molar ratio of 1.0. Both interactions were exothermic, characterized by negative enthalpy changes; however, the enthalpy change associated with the benzoic acid–stearic acid interaction was significantly greater. Considering the corresponding entropy changes, the study concluded that the benzoic acid–stearic acid interaction was predominantly driven by hydrogen bonding, whereas the salicylic acid–stearic acid interaction was mainly governed by hydrophobic forces [51]. Although this study focused on specific molecules and its conclusions may not be universally applicable to all hydrophobic compounds, it demonstrated the efficacy of ITC in quantitatively characterizing molecular interactions within solid lipid nanoparticles. This approach can be extended to study interactions between various drug molecules and lipid components. Moreover, ITC has also been employed to investigate drug localization within solid lipid nanoparticles. For example, the binding of thionine, a cationic phenothiazinium dye, to mRNA was studied by ITC as a model system to probe drug permeation and localization inside solid lipid nanoparticles [52].

Most solid lipid nanoparticles are composed of multiple components [53], which complicates the interpretation of specific molecular interactions by ITC. Consequently, many ITC studies on solid lipid nanoparticles focused on protein adsorption, commonly referred to as the formation of the “protein corona”, using various proteins as model systems. For example, Rathee et al. investigated how ITC has been applied to investigate the interaction between solid lipid nanoparticles and bovine serum albumin (BSA) by titrating BSA into a solid lipid nanoparticle suspension [54]. Due to nonspecific and very weak interactions between BSA and the nanoparticles, the resulting ITC thermograms lacked distinct binding profiles and showed very low heat rates. As a result, no meaningful thermodynamic parameters could be extracted. The observed interactions were likely driven by hydrophobic interactions between hydrophobic patches on the BSA surface and the lipid nanoparticles [54]. Similarly, another ITC study by Miao et al. demonstrated that the interaction between human serum albumin (HSA) and solid lipid nanoparticles was weak but the interactions were dependent on lipid composition [55]. In this work, alkyne and ester groups were incorporated into the lipid tails of Dlin-MC3-DMA, an ionizable cationic lipid containing a tertiary amine group that becomes protonated under acidic conditions and is widely used in clinically approved lipid nanoparticle formulations. The solid lipid nanoparticles were co-formulated with fusogenic lipids such as dioleoylphosphatidylethanolamine (DOPE) and compared with formulations containing unmodified Dlin-MC3-DMA or cKK-E12, a tripeptide-derived ionizable cationic lipid designed for efficient mRNA delivery to hepatocytes. ITC measurements revealed that solid lipid nanoparticles containing lipids with alkyne and ester modifications exhibited a fivefold lower association constant for HSA adsorption compared to cKK-E12 nanoparticles. This reduced HSA binding correlated with significantly enhanced mRNA delivery to the liver [55]. Another similar study investigated the effects of particle size and pH on BSA adsorption to model lipid nanoparticles composed of cetyl palmitate and Tween 80 [56]. At pH 7.4, ITC thermograms indicated exothermic interactions (ΔH < 0) coupled with negative entropy changes (ΔS < 0), suggesting that van der Waals forces and hydrogen bonding dominated the interaction [32]. In contrast, at pH 6.0, the ITC profiles exhibited both endothermic and exothermic phases, implying a more complex interplay of interaction forces, including electrostatic and hydrophobic interactions alongside van der Waals forces and hydrogen bonding. Furthermore, smaller lipid nanoparticles adsorbed less BSA, highlighting size-dependent protein corona formation.

Beyond serum albumins, apolipoprotein A1 (ApoA1) is another critical protein that modulates nanoparticle delivery. ApoA1 is the major protein component of high-density lipoprotein and its interaction with lipid-based nanocarriers can impact the stability, cholesterol efflux, and overall efficacy of the nanocarrier [57]. An ITC study examined the ApoA1 adsorption onto PEGylated lipid shell-coated nanoparticles with varying elasticity [58]. These nanoparticles included empty liposomes composed of 1,2-dioleoyl-sn-glycero-3-phosphoethanolamine (DOPC) and DSPE-PEG2000 (soft), lipid shells with acrylamide hydrogel cores (intermediate elasticity), and lipid shells with poly(lactic-co-glycolic acid) (PLGA) cores (stiff). ITC thermograms showed a stronger ApoA1 binding to nanoparticles of intermediate elasticity while soft and stiff nanoparticles showed no ApoA1 adsorption [58]. In contrast, BSA adsorption was unaffected by nanoparticle elasticity. This study underscores the synergistic role of ApoA1 and nanoparticle elasticity in modulating the systemic circulation lifetime of lipid nanoparticles.

Although these ITC studies were conducted using various model lipids and proteins without a unified conclusion, the ITC results collectively demonstrated that ITC is a powerful technique for investigating molecular interactions involving solid lipid nanoparticles. However, the complexity of solid lipid nanoparticle compositions can complicate data interpretation, where careful experimental design, proper data fitting, and thorough background correction need to be ensured for reliable results.

### 3.2. ITC Applications in Liposomes

Liposomes, also known as lipid bilayer vesicles, membrane vesicles, or lipid vesicles, are a highly promising class of biomimetic nanocarriers for drug delivery applications [4,59]. Their unique bilayer structures enable the encapsulation of both hydrophilic and hydrophobic agents, including small-molecule drugs, proteins, RNA, and DNA. Compared to solid lipid nanoparticles, liposomes feature a characteristic hollow core surrounded by a lipid bilayer, which provides both versatility and biocompatibility [60]. The lipids used to form liposomes can be natural and/or synthetic, commonly including phospholipids such as phosphatidylcholine (PC), phosphatidylethanolamine (PE), phosphatidic acid (PA), phosphatidylserine (PS), 1-palmitoyl-2-oleoyl-sn-glycero-3-phosphocholine (POPC), 1-palmitoyl-2-oleoyl-sn-glycero-3-phospho-(1’-rac-glycerol) (POPG), DOPC, DOPE, 1,2-dimyristoyl-sn-glycero-3-phosphocholine (DMPC), cardiolipin (CL), and 1,2-dioleoyl-3-trimethylammoniumpropane (DOTAP). ITC has emerged as a powerful technique for studying molecular interactions involving liposomes. ITC enables detailed thermodynamic characterization of liposome interactions with a wide range of components, including drug molecules, peptides, proteins, and other macromolecules. Furthermore, ITC can be employed to assess liposome stability and their interactions with biological systems, offering valuable insights into cellular uptake and circulation behavior. For example, ITC has been effectively employed to study how lipid composition and dipole orientation influenced the properties of liposomes [61]. The following section will elucidate the applications of ITC in characterizing structural properties of liposomes and investigating their interactions with various biomolecules.

### 3.3. Membrane Solubilization and Stability

Membrane solubilization and stability are two critical factors in the design and performance of liposomal drug nanocarriers, as they directly influence drug loading capacity, release kinetics, and overall therapeutic efficacy. These properties can be finely tuned through the incorporation of additional molecules, such as surfactants [60]. Surfactants are commonly used for the extraction of membrane proteins, providing valuable insights into protein structure and function [62]. Beyond protein extraction, surfactant-like molecules integrated into lipid bilayers can serve as powerful modulators of membrane properties. For instance, cholesterol functions as a molecular “glue” within the lipid bilayer, enhancing membrane stability and regulating bilayer permeability. Cholesterol inclusion in liposomes has been shown to significantly alter encapsulation efficiency and drug release profiles of liposomes [63]. These structural modifications to the lipid membrane are typically accompanied by changes in system energetics, which can be directly quantified using ITC [64]. The resulting thermodynamic data can be correlated with lipid composition and structural characteristics, providing critical insights for the rational design of stable and efficient liposomal formulations.

**Figure 5:**
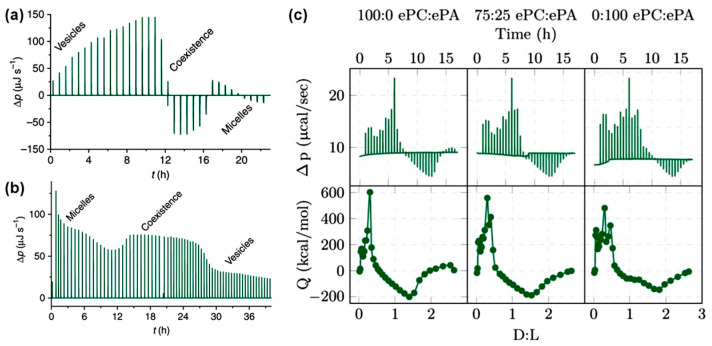
(**a**) Representative ITC thermograms showing the titration of the nonionic surfactant C10EO5 into POPC lipid vesicles. The data illustrate a transition from an initial endothermic process (associated with surfactant insertion into lipid bilayers) to an exothermic process (corresponding to micelle formation) as surfactant concentration increases. (**b**) ITC thermograms showing the titration of POPC lipid vesicles into C10EO5 solution, illustrating the vesicle reconstruction process. (**c**) ITC thermograms and corresponding injection heats for titrations of Triton X-100 into liposomes composed of pure PC, a 3:1 molar ratio of PC:PA, and pure PA. The data reveal distinct solubilization behaviors and energetic profiles dependent on lipid composition. (**a**,**b**) Reproduced with permission from [69], Springer Nature, Copyright 2009. (**c**) Reproduced with permission from [70], Elsevier, Copyright 2020.

The solubilization and reconstitution of liposomes by surfactants or surfactant-like molecules play a crucial role in various technical applications, such as the isolation of membrane proteins [65]. Surfactant-induced membrane solubilization is typically described by a well-established three-stage model: (1) insertion of surfactants into intact lipid vesicles, (2) coexistence of vesicles and mixed micelles, and (3) complete solubilization of vesicles, resulting in the formation of mixed micelles composed of lipids and surfactants [66]. The transitions between these stages depend strongly on the molecular shapes of both the lipids and the surfactants involved. These transitions are also accompanied by measurable energy changes that can be quantitatively analyzed using ITC [65]. Quantitative data on surfactant concentrations required for membrane solubilization and stabilization provide valuable insights into bilayer structural design. Therefore, ITC serves as an effective tool to investigate surfactant-induced solubilization and reconstruction of liposomes [67,68]. At low concentrations, surfactants typically insert partially or fully into lipid bilayers. Depending on the surfactant’s molecular structure, this insertion process can be either endothermic or exothermic. As surfactant concentration increases, the lipid membrane undergoes solubilization, leading to the formation of micelle-like structures. For example, Figure 5a shows a representative ITC thermogram obtained by titrating the nonionic surfactant alkyl polyethylene glycol ether (C10EO5) into POPC lipid vesicles. The thermogram clearly demonstrates the transition from an endothermic surfactant insertion to an exothermic micelle formation [69]. Notably, the total heat released during these reactions depends on the reactant concentrations, even when the surfactant-to-lipid ratio remains constant. A similar ITC study was also performed by titrating hexaethylene glycol mono-n-dodecyl ether (C12EO6) into POPC liposomes, which showed surfactant insertion into membranes up to a certain concentration followed by membrane solubilization into micelles. The ITC thermograms also exhibited a characteristic transition from endothermic to exothermic behavior [68]. The ITC study also allows flexibility in experimental design depending on the titration order. Titrating surfactants into membrane vesicles enables real-time monitoring of membrane solubilization. Conversely, titrating lipid vesicles into a surfactant solution provides insight into membrane reconstitution dynamics (Figure 5b) [69]. Compared to the membrane solubilization studies, no distinct transition was observed in the thermograms; however, the magnitude of the power changes clearly indicated structural alterations. These alternative approaches offer complementary thermodynamic information for the same lipid-surfactant system, enhancing our understanding of membrane remodeling processes.

Furthermore, an ITC investigation involving titration of Triton X-100, a common non-ionic surfactant, into liposomes composed of mixed egg lipids (PC and PA) highlighted the direct influence of lipid composition on the solubilization behavior of liposomes [70]. The ratio of PC to PA was found to affect the lipid packing within the liposomes and subsequently modulate surfactant partitioning prior to liposome disruption and micelle formation. The ITC thermograms distinctly captured surfactant partitioning into the lipid bilayers and micelle formation at high Triton X-100 concentrations. These two processes, saturation and solubilization, exhibited characteristic endothermic or exothermic signals, with the transition dependent on lipid composition, as shown in Figure 5c. ITC data further indicated that liposomes composed entirely of PA required higher Triton X-100 concentrations for membrane solubilization compared to those made purely of PC, while mixed lipid liposomes containing 25% PA exhibited solubilization behavior liposomes more similar to that of liposomes composed entirely of PA [70]. In addition to lipid composition, liposome curvature also impacted membrane solubilization behavior. A similar ITC study titrating Triton X-100 into DMPC vesicles demonstrated that liposomes with higher curvature exhibited increased reactant enthalpy, suggesting curvature-dependent modulation of solubilization energetics [71].

In addition to lipid composition, the thermodynamic profile, whether endothermic or exothermic, observed during the titration of surfactants into liposomes also depends on the types of surfactants used. Transitions from endothermic to exothermic behavior are particularly characteristic of titrating nonionic surfactants into liposomes. In contrast, ionic surfactants often display distinct interaction profiles. An ITC study by Keller et al. [72] investigated the solubilization and reconstitution of POPC membrane vesicles using the anionic surfactant sodium dodecyl sulfate (SDS). Unlike the typically endothermic partitioning observed with nonionic surfactants, the insertion of dodecyl sulfate anions into POPC bilayers was found to be an exothermic process. Furthermore, the enthalpy of SDS partitioning into the membrane decreased linearly with increasing temperature, highlighting the temperature sensitivity of ionic surfactant–lipid interactions.

Similarly to surfactant insertion, other surfactant-like molecules can also integrate into lipid membranes and modulate their properties [73]. One well-known example is cholesterol, which intercalates between phospholipid molecules within the liposomal bilayer. This incorporation increases membrane packing density and enhances membrane integrity, particularly under stress conditions such as prolonged storage or circulation in the bloodstream. In addition to improving stability, cholesterol also reduces the permeability of the bilayer to small, water-soluble molecules. This property is especially important for retaining hydrophilic drugs within liposomes and for controlling the rate of drug release. Liu et al. [74] investigated the effects of cholesterol on the size of PC lipid vesicles and their response to mechanical stress. Their study showed that cholesterol incorporation increased the rigidity of the vesicles, resulting in stronger repulsive interactions between them and enhanced resistance to shear forces. These findings underscore the crucial role of cholesterol in improving both the physical stability and functional performance of liposomal drug carriers.

Similarly, poloxamers, amphiphilic triblock copolymers, can interact with liposomes and effectively partition into the lipid bilayer, a process that has been studied by ITC [75]. Like surfactants, poloxamers at low concentrations can insert into lipid bilayers without disrupting vesicle integrity. However, at higher concentrations, they can destabilize and disintegrate liposomes into micelle-like structures. In an ITC study, the interactions of two block copolymers, F108 and F127, with lipid vesicles at physiological temperature were examined [76]. Both F108 and F127 are pluronic polymers (also known as poloxamers), which are amphiphilic triblock copolymers. Both have the same general structure, but F108 has a higher proportion of hydrophilic blocks compared to its hydrophobic block and F127 has a larger hydrophobic block than F108. The interactions of both polymers with lipid vesicles were found to be endothermic, in contrast to the typical exothermic behavior observed with many ionic surfactants. Of the two, F127 exhibited a higher partition coefficient and stronger affinity for the lipid bilayer than F108, attributed to its longer hydrophobic block. These findings underscore that both the concentration and the hydrophobic chain length of poloxamers significantly influence their interaction with lipid membranes and the resulting liposome stability. Additionally, the structure of small amphiphilic molecules, such as alkanols, can modulate membrane stability and integrity [77]. ITC studies have shown that both the molecular shape (linear vs. branched) and chain length of alkanols affected their interactions with phospholipid bilayers [77]. For example, branched alkanols displayed lower binding affinity compared to their linear counterparts. Short-chain alcohols (e.g., ethanol with fewer than six carbon atoms) primarily interacted at the bilayer interface and induced greater enthalpic changes. In contrast, longer-chain alcohols such as octanol exhibited restricted bilayer penetration due to their size, resulting in reduced enthalpic effects compared to smaller alcohols like hexanol. Such thermodynamic insights are particularly useful when considering the incorporation of short-chain alcohols as penetration enhancers in liposomal formulations.

In addition to cholesterol and surfactant-like molecules, the interactions of charged components, such as anionic or cationic lipids or charged macromolecules, can also enhance liposome stability [73]. These charged components generate electrostatic repulsion between liposomes, effectively preventing aggregation and promoting colloidal stability. Moreover, the biophysical properties of liposomes can be further modulated through interactions with polymeric molecules [78]. For instance, ITC has been used to study the interactions between negatively charged liposomes and cationic dendrimers of various generations [79]. The ITC results revealed that the electrostatic interactions between dendrimers and lipid bilayers were exothermic and dependent on the dendrimer generation, with higher generations exhibiting stronger binding due to increased surface charge density. Similarly, ITC analysis of the interactions between chitosan, a cationic polysaccharide, and liposomes demonstrated strong electrostatic attraction between chitosan and negatively charged membranes, producing highly exothermic signals [80]. These interactions contributed to the formation of stable liposome suspensions. Thermodynamic parameters such as the equilibrium constant, binding stoichiometry, and molar enthalpy of binding were obtained by fitting the isotherm curves under the assumption of independent binding sites. The results also showed distinct enthalpy changes depending on whether chitosan interacted with neutral or negatively charged liposomes, emphasizing the importance of membrane charges in determining binding strength. Furthermore, an ITC study investigating the interaction between DOPC liposomes and cellulose nanocrystals reported an endothermic process that appeared independent of lipid concentration [81]. This study suggested that non-electrostatic factors, such as hydrogen bonding and hydrophobic interactions, may also contribute to the overall thermodynamics of lipid-polymer interactions. Through careful adjustment of experimental conditions, ITC can also be effectively employed to study interactions involving molecules with relatively low binding affinity [82].

In summary, the properties and performance of liposomes depend on an optimal balance between membrane solubilization and structural stability. Excessive solubilization, for example, due to high concentrations of surfactants, can compromise the integrity of the lipid bilayer, resulting in leaky vesicles and reduced drug retention. Conversely, overly rigid membranes may hinder drug release and limit cellular uptake. Therefore, maintaining a balance of solubilization and stability is critical to ensure efficient drug encapsulation, sustained release profiles, and structural integrity in physiological environments.

### 3.4. Cargo Loading into Liposomes

The structural features of liposomes enable the encapsulation of both hydrophilic and hydrophobic drugs. Typically, hydrophobic drugs are incorporated into the lipid bilayer, whereas hydrophilic drugs must translocate across the lipid membrane to reach the aqueous interior compartment [83]. ITC protocols have been developed to study membrane partitioning, translocation, and release of various compound classes by measuring membrane binding and translocation behavior of compounds [83,84]. In typical ITC experiments involving liposomes, either the test compound is titrated into the liposome suspension, or the liposomes are titrated into the compound solution, depending on the experimental objective. For instance, Osanai et al. [85] investigated the interactions between several model drug molecules and PC liposomes using ITC, by titrating liposomes into drug-containing solutions. This experimental design ensured an excess of drug molecules relative to the number of liposomes, allowing detailed assessment of binding behavior. The drugs tested in this study included ANS (1-anilino-8-naphthalenesulfonate), TPB (tetraphenylborate), amlodipine, nifedipine, amitriptyline, nortriptyline, imipramine, desipramine, propranolol, chlorpromazine, promethazine, miconazole, indomethacin, diclofenac, and diflunisal. These 15 compounds were selected to provide a broad range of physicochemical characteristics, including variations in aromaticity, molecular size, hydrophobicity, charge, and hydrogen-bonding capacity. Such diversity enabled systematic evaluation of how distinct molecular features govern partitioning, binding thermodynamics, and membrane destabilization potential. The resulting ITC thermograms and derived thermodynamic parameters suggested that the interactions between many of the tested drugs and liposomes were primarily entropy-driven, indicative of hydrophobic interactions [85]. However, for less hydrophobic drugs such as TPB, ANS, amlodipine, desipramine, and diflunisal, the enthalpy change was the dominant thermodynamic contribution. These findings demonstrated that the nature and strength of drug-liposome interactions were highly dependent on the structural and physicochemical properties of the drug molecules [85].

ITC studies can further provide valuable insights into the localization and interactions of drug molecules with varying polarities within liposomal membranes [86]. For example, an ITC study investigated the interactions of three drugs, erythromycin (a macrolide antimitotic), kanamycin (an aminoglycoside antibiotic), and 5-fluorouracil (a chemotherapeutic agent), with liposomes composed of DPPC, DSPC, and cholesterol [86]. Due to its hydrophobic nature and bulky structure, erythromycin failed to produce a measurable ITC thermogram, suggesting poor solubility or minimal interaction with the aqueous environment of the liposomes. In contrast, the ITC thermograms for kanamycin and 5-fluorouracil interactions with liposomes exhibited distinct profiles. Although both compounds are hydrophilic, 5-fluorouracil showed an endothermic interaction, whereas kanamycin displayed an exothermic response [86]. The data for both compounds were best fitted using a sequential two-site binding model. The ITC thermogram for kanamycin revealed an overall exothermic process, characterized by an initial endothermic event followed by a smaller exothermic signal. Kanamycin also demonstrated stronger partitioning into liposomes compared to 5-fluorouracil. This process was found to be entropy-driven, as evidenced by the decreasing entropy change from the first to the second binding site [86]. In addition to drug properties, the drug-to-lipid ratio significantly influenced drug–lipid interactions. For instance, ITC was used to characterize and quantify the partitioning of two nonsteroidal anti-inflammatory drugs, indomethacin and acemetacin, into PC liposomes [87]. The ITC thermograms indicated exothermic interactions for both drugs, with electrostatic forces playing a significant role across all concentrations and lipid/drug ratio ratios. However, the magnitude of heat release depended strongly on the lipid/drug ratio: enthalpy was constant above ~100:1 but varied and then stabilized at lower ratios. Such lipid to drug ratios (~50:1 to 200:1) are typical for liposomal formulations and are consistent with values reported for clinically relevant systems such as Doxil^®^, where excess lipid ensures drug encapsulation stability and minimizes premature release [88].

Furthermore, ITC was utilized to characterize molecular interactions between bioactive compounds and liposomes [89]. In this study, the influence of liposome composition on both liposome formation and their interactions with bioactive compounds from citrus extract and essential oils was examined. ITC analysis showed that liposomes exhibited greater affinity for encapsulating citrus extract compared to essential oils. This was likely due to increased surface contact between the hydrophilic components of citrus extract and the aqueous core of the vesicles, enhancing the binding affinity. Another ITC study of drug–liposome interaction was performed to evaluate the binding of the traditional Chinese medicine *Jingui Shenqi* to DPPC liposomes [90]. In this study, different drug components, using cinnamaldehyde and loganin as representative indicators, were titrated into DPPC vesicles. The ITC thermodynamic data provided insights into the nature and strength of these drug–membrane interactions, further supporting ITC as a powerful tool for characterizing the behavior of complex drug formulations with lipid bilayers.

In addition to drug molecules, ITC can be effectively used to study the incorporation of other functional compounds into liposomal membranes. For example, ITC was employed to investigate the association of Hoechst 33342, a fluorescent dye commonly used for live-cell DNA staining, with POPC lipid bilayers [91]. The enthalpy obtained from ITC data fitting revealed that the interaction of Hoechst 33342 with POPC membranes was predominantly enthalpy-driven at pH 5.3. In contrast, at pH 8.2, both enthalpic and entropic contributions were found to be equally significant. These results suggest that at higher pH, the interaction was primarily mediated by hydrophobic forces involving the neutral form of Hoechst 33342, while at lower pH, electrostatic interactions and hydrogen bonding involving the cationic form of the dye dominated the binding mechanism [91]. Beyond small molecules, ITC has also been applied to investigate the interactions between PC liposomes and nanoparticles, particularly in the development of stimulus-responsive liposomal systems. For instance, carbon-based nanoparticles have been incorporated into liposomes to create photo responsive delivery platforms [92]. These ITC studies provide valuable thermodynamic insights into nanoparticle-lipid interactions, supporting the rational design of advanced functional liposomes for targeted and controlled drug release.

### 3.5. Non-Specific Interactions of Liposomes

In the context of peptide drug delivery using liposomes, ITC studies primarily focused on two key areas: the interactions of liposomes with antimicrobial peptides and the membrane-associating behavior of cell-penetrating peptides.

**Figure 6:**
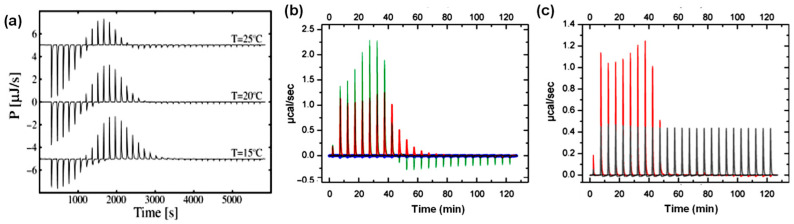
(**a**) ITC thermograms of POPC/POPG (1:3) lipid vesicles titrated into a mastoparan-X solution at 15, 20, and 25 °C, showing temperature-dependent thermodynamic profiles. (**b**) ITC thermograms of lipid vesicles titrated into Pln149 solution: POPC (blue), POPG (red), and POPS (green), highlighting the influence of lipid composition on peptide interactions. (**c**) ITC thermograms of POPG vesicles titrated into Pln149 solution in the absence (red) and presence (gray) of 250 mM NaCl, illustrating the effect of ionic strength on binding behavior. (**a**) Reproduced with permission from [95], Elsevier, Copyright 2011, (**b**,**c**) Reproduced with permission from [96], Springer Nature, Copyright 2019.

Antimicrobial peptides are typically cationic molecules that interact strongly with negatively charged lipid membranes, primarily through electrostatic interactions. Upon initial insertion into the lipid membrane of liposomes, some antimicrobial peptides can form pores within the lipid bilayer [93]. This process is often followed by membrane solubilization and the formation of mixed peptide–lipid micelles [93]. Such membrane-disrupting mechanisms have proven to be an effective strategy for combating multidrug-resistant bacteria [93] ITC has been extensively used to quantify the binding affinity of antimicrobial peptides to lipid bilayers, enabling direct comparisons of peptide efficiency and the contribution of specific amino acid residues of peptide drugs [94]. ITC has been shown to be highly effective at probing the energetics of the entire peptide–membrane interaction process, including initial binding, insertion, and pore formation [95]. For example, in an ITC study of titrating POPC/POPG (3:1) liposomes into the antimicrobial peptide, mastoparan-X, solution, a clear exothermic-to-endothermic transition was observed [95], where the trend of the thermograms was quite different from titrating liposome into surfactant solution, as shown in Figure 6b. By adjusting the temperatures of the ITC experiments, a key metric can be derived from data analysis, defined as zero enthalpy temperature (T_zero_), at which the enthalpy of peptide partitioning became zero. Determining T_zero_ through ITC data analysis allowed for precise characterization of the thermodynamics involved in both the formation and disintegration of membrane pores (Figure 6a) [95]. A similar ITC study investigated the interactions between another antimicrobial peptide, Pln149, and model membranes composed of zwitterionic and/or anionic phospholipids [96]. The ITC results demonstrated that peptide-lipid interactions may arise from a combination of electrostatic attraction between cationic peptides and negatively charged membranes, peptide insertion into the polar headgroup region or hydrophobic core, and conformational changes such as pore formation. The ITC binding thermograms of Pln149 with anionic POPG vesicles (Figure 6b, red) showed an endothermic process [96]. The reaction enthalpy (ΔH) gradually decreased until the heat of partitioning plateaued, indicating potential saturation of peptide-membrane binding sites. Similar behavior was observed with anionic POPS vesicles (Figure 6b, green), although the enthalpy of partitioning was approximately twice as high as that for POPG, suggesting stronger interactions. In both cases, the initial increase in heat release, followed by a gradual decrease to a baseline, indicated a possible multi-binding process. In contrast, no significant heat change was detected when Pln149 interacted with zwitterionic POPC vesicles (Figure 6b, blue), highlighting the importance of membrane charge in cationic peptide binding. Furthermore, the presence of NaCl significantly reduced heat generation (Figure 6c), consistent with the disruption of electrostatic interactions under high ionic strength conditions [96].

Additionally, ITC was employed to study interactions between a synthetic antibacterial peptide, TxI mn∆K, derived from spider venom, and liposomes composed of POPG, CL, and 1,2-dioleoyl-sn-3-lysyl(1-glycerol) (Lysyl) [97]. In these experiments, liposome suspensions were titrated into peptide solutions or large membrane vesicle suspensions to mimic cell membranes. The resulting enthalpy changes indicated an exothermic interaction that quickly reached equilibrium. Thermodynamic analysis suggested that both enthalpic and entropic contributions played roles in the peptide-membrane binding process. Similarly, ITC was used to study the membrane interactions of the antimicrobial peptide cR3W3 with various model membranes, including POPG/POPE, POPG/POPC, and POPG/POPE/DSPE-PEG2000 [98]. The ITC thermograms showed consistent exothermic binding across all systems, with no substantial differences in binding affinities. However, notable differences were observed in binding stoichiometry where cR3W3 bound to POPG/POPE membranes with a stoichiometry of approximately 4.5 lipids per peptide, compared to 6.1 lipids per peptide for POPG/POPC membranes, suggesting variations in membrane composition can influence peptide packing and interaction density.

Beside antibacterial peptide, the interactions of liposomes with exenatide, a tryptophan-containing, cationic peptide drug used in the treatment of diabetes, were also investigated using ITC [99]. Theoretically, cationic exenatide is expected to adsorb onto anionic liposomes, such as POPG, via electrostatic interactions. However, the obtained ITC thermograms were complex and could not be accurately fitted using conventional binding site models. This complexity likely resulted from multifaceted interactions between exenatide and lipid membranes. It was proposed that exenatide could initially interact with membrane through electrostatic attractions, and subsequently, the adsorbed peptides on liposome surfaces could act as secondary binding sites for additional peptide molecules. When primary and secondary binding events were analyzed separately, ITC data indicated that secondary binding was characterized by weaker affinity and a less exothermic, or even endothermic, thermodynamic signature [99]. Similarly, ITC was also employed to investigate the association of the endogenous heptapeptide, VV-hemorphin-5 (valorphin), with POPC bilayers [100]. The strength of valorphin–membrane association was found to be concentration-dependent, with stronger binding observed at higher peptide concentrations. This enhanced association correlated with a greater impact on the mechanical properties of the lipid bilayer, indicating that valorphin can modulate membrane characteristics in a dose-dependent manner.

Another important category of peptide-liposome interactions involves liposome functionalization by cell-penetrating peptides (CPPs), which are widely used as therapeutic and diagnostic delivery tools [101]. CPPs are typically cationic and capable of efficient cellular uptake, making them ideal candidates for intracellular drug delivery [102]. The binding of these peptides to lipid membranes, as well as their cellular interactions, can be effectively characterized using ITC [102]. For instance, Ziegler and colleagues [103]. employed ITC to investigate the binding of TAT peptides to anionic POPG membranes. TAT, a well-known CPP derived from the transactivator of transcription (Tat) protein of HIV-1, interacts predominantly through electrostatic attraction with negatively charged membranes. Similarly to cationic peptides, polycationic polymers such as polyarginine and polylysine also exhibit strong interactions with anionic lipid membranes [104]. ITC studies demonstrated that while both polymers can penetrate lipid bilayers, polyarginine exhibited approximately twice the binding enthalpy compared to polylysine. This study suggested that arginine residues interacted more strongly with negatively charged membranes, likely due to differences in the conformational behavior of the polypeptides during membrane insertion [104].

Beyond electrostatic interactions of cationic peptides with liposomes, liposomes can interact with molecules through other types of weak interactions. For example, ITC has been applied to investigate membrane fusion events where liposomes composed of equimolar amounts of the cationic lipid DOTAP and the fusogenic phospholipid DOPE were shown to undergo efficient fusion with model vesicles made of zwitterionic POPC [105]. The fusion process was exothermic in the absence of cholesterol, indicating that electrostatic interactions were the dominant driving force. However, as cholesterol content increased, particularly beyond 30%, an endothermic component emerged and eventually dominated, suggesting a shift in the fusion mechanism. These results highlighted that membrane fusion efficiency in the DOTAP/DOPE system was modulated by both membrane charge and packing, reflecting conditions similar to those found in biological membranes [105]. Similarly, the interactions between a bioactive peptide, Asn-Cys-Tr, and DPPC liposomes was investigated using ITC [106]. The thermodynamic data suggested that the binding was predominantly entropy-driven and involved electrostatic forces, hydrogen bonding, and hydrophobic interactions. ITC has also been used to study the interaction of amyloid beta (Aβ)-(1–40) monomers with DOPC liposomes of two distinct sizes (30 nm and 100 nm), which serve as models for membranes with high and low curvature, respectively [107]. The ITC thermograms revealed that interactions with 100 nm liposomes were exothermic, while those with 30 nm liposomes were endothermic and associated with larger heat changes. Thermodynamic analysis further indicated a higher binding affinity of Aβ monomers for the 100 nm liposomes. These results suggest that membrane curvature significantly influences Aβ binding behavior, which in turn affects aggregation pathways and fibrillation kinetics [107].

In addition to peptide–liposome interactions, ITC has also been widely used to study protein-liposome interactions [108,109,110]. Using BSA as a model protein, ITC studies revealed that lipid composition significantly influences membrane properties such as deformability, lipid intermixing, and the formation of lipid domains. Furthermore, BSA binding to the liposome surface was modulated by the presence of PEGylated lipids and cholesterol [111]. These results underscore the critical role of lipid composition in governing protein–liposome interactions. Additionally, ITC has also been employed to study the association of calcium ions (Ca^2+^), a representative ion commonly found in serum, with lipid bilayers, as a function of vesicle composition and preparation method [112]. The heat changes observed upon titrating Ca^2+^ solution into a vesicle-containing buffer indicated that the binding enthalpy of Ca^2+^ to lipid vesicles composed of POPC and POPG was highly dependent on the method used to prepare the vesicles. Specifically, vesicles prepared by extrusion exhibited an exothermic interaction with Ca^2+^, whereas those prepared via sonication showed an endothermic binding profile. These contrasting thermodynamic signatures are likely attributable to differences in vesicle size and structural organization resulting from the two preparation techniques. As expected, the interactions between Ca^2+^ and the negatively charged phospholipid bilayers were primarily electrostatic in nature. Furthermore, increasing the ionic strength of the buffer led to a decrease in the binding affinity, consistent with ionic screening effects that weaken electrostatic interactions.

Although most of these reported ITC studies were based on non-specific interactions of molecules with liposomes, the thermodynamic parameters obtained from different systems offer valuable insights for the rational design of liposomes in drug delivery applications

### 3.6. Specific Interactions with Liposomes

Beyond nonspecific interactions between liposomes and macromolecules, ITC can also be employed to investigate specific interactions of membrane proteins reconstituted into lipid bilayers or liposomes. For example, multidrug transport proteins incorporated into liposomes have been studied using ITC to assess the effects of lipid composition and to quantify the recovery of functional protein under different reconstitution conditions [113]. Reconstituting membrane proteins into liposomes provides a biologically relevant environment that facilitates the study of their binding behaviors and functional mechanisms. The binding of ligands to membrane proteins is typically associated with measurable heat changes, either endothermic or exothermic, which can be sensitively detected by ITC. This technique has been applied to characterize the functional properties of the small multidrug resistance protein, EmrE, from *Escherichia coli* (*E. coli*), reconstituted into lipid vesicles [113]. The proportion of properly folded and functional EmrE proteins within liposomes was found to depend on the lipid composition of the vesicles. ITC measurements allowed for direct quantification of substrate binding, thereby confirming the functional states of the reconstituted protein [113]. Similarly, ITC has been explored as a valuable tool to study ion-coupled membrane transporters [114]. In this study, ITC thermograms and data fitting were used to evaluate the binding of chloride ions (Cl^−^) and protons (H^+^) to the Cl^−^/H^+^ antiporter protein. The ITC analysis not only revealed the number of coupled ions transported but also elucidated the mechanism of coupling between the ions and their substrates [114]. Although only a limited number of studies have been reported for specific interactions using liposomes, these investigations have clearly demonstrated the feasibility of using ITC to quantitatively assess such interactions.

In summary, ITC is a powerful technique for elucidating the molecular mechanisms by which ions, drugs, peptides, and proteins interact with liposomal membranes. The thermodynamic insights are essential for optimizing liposome-based drug delivery systems and improving the design of lipid-based nanocarriers.

### 3.7. ITC Applications of Cell-Derived Vesicles

Compared to the well-studied solid lipid nanoparticles and liposomes, cell-derived membrane vesicles are gaining increasing attention as promising nanocarriers for drug delivery, such as EVs and cell membrane-coated nanoparticles (also known as nanoghosts). EVs are naturally occurring, nanoscale membrane-bound vesicles released by cells as part of intercellular communication processes [115,116,117]. In contrast, nanoghosts are engineered nanocarriers, consisting of a nanoparticle core for drug encapsulation and a shell of cell membrane derived from cells of interests [118,119]. Both EVs and nanoghosts retain signature transmembrane proteins from their parental cells, which offer unique biological functions [120,121], such as prolonged blood circulation [122], tumor targeting [119,123], and the ability to cross biological barriers [124]. The nanoghost platform is particularly robust and versatile and has been explored for a wide variety of membrane types and nanoparticle cores as potential drug delivery vehicles [118,119,125,126]. Despite the growing interest in both EVs and nanoghosts for drug delivery applications, the use of ITC to investigate their molecular interactions with biological systems remains very limited.

**Figure 7:**
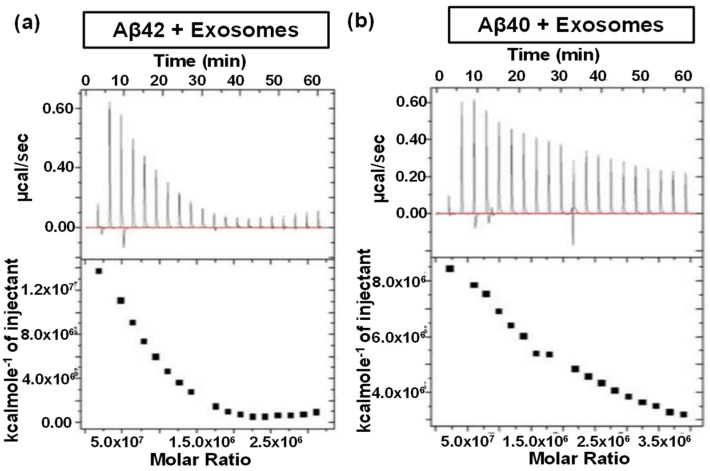
ITC thermograms of Aβ binds to EVs by titration EV solution into (**a**) Aβ40 and (**b**) Aβ42 solution. Reproduced from [127] CC-BY 4.0.

The importance of EVs in biological systems is closely related to their distinct surface protein markers, such as tetraspanins (e.g., CD9 and CD63) [113], which play a critical role in their interactions with biological systems. Additionally, EVs carry specific membrane proteins upregulated on parental disease cells, such as P-glycoprotein (Pgp) efflux pumps commonly found on drug-resistant cancer cells [114]. A comprehensive understanding of EV molecular interactions is crucial for full realization of EV potentials such as drug delivery vehicles and diagnostic tools. Theoretically, ITC offers a powerful and versatile approach for characterizing a range of EV-related molecular interactions, such as ligand-protein marker interactions, cellular uptake behaviors of EVs through membrane fusion like liposome systems. However, the inherent heterogeneity of EVs poses significant challenges. Therefore, only one ITC study on EVs was found to investigate the specific binding interactions between Alzheimer’s amyloid-β (Aβ) peptides and EVs [127]. In this study, EVs isolated from human plasma were titrated into Aβ42 or Aβ40 peptide solutions of to characterize their molecular interactions. The resulting ITC thermograms (Figure 7) suggested that both Aβ42 and Aβ40 bind to EVs in a sequential, specific, and saturable manner. In addition, the interactions were endothermic, indicating an entropy-driven binding process. These findings suggest a potential physiological role for EVs in the transport of amyloid peptides from the brain to the bloodstream.

Although the intrinsic heterogeneity of EVs presents challenges in obtaining precise quantitative thermodynamic parameters, ITC thermograms offer a valuable opportunity to compare the direct binding behaviors of EVs with various biomolecules. In contrast to well-characterized systems such as liposomes and solid lipid nanoparticles, a key distinguishing feature of EVs is the presence of diverse protein markers on their surfaces. By accurately quantifying both the EV concentration and the specific surface biomarkers, ITC has the potential to reveal the thermodynamics of ligand or drug interactions with EV surface proteins. The reliability of the derived thermodynamic parameters will depend on the precision of surface protein quantification. To distinguish specific from nonspecific binding, EVs expressing different surface markers can be used as comparative controls. While direct ITC studies on EVs remain limited, methodologies developed for liposome-based systems and membrane proteins [23,119] can potentially be extended to EVs. For instance, transmembrane proteins reconstructed within liposomes have been used to study protein-ligand interactions effectively using ITC [104], which may be translated to study ligand–biomarker interactions on EV surfaces. Additionally, studies focusing on membrane stability and solubilization may help mimic engineered EVs for drug delivery applications.

**Figure 8:**
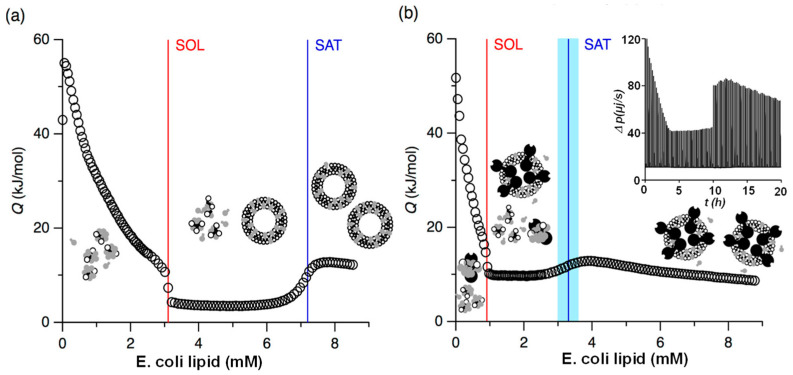
ITC reconstitution isotherms in (**a**) the absence and (**b**) the presence of 1.5 μM KcsA tetramer at 8 °C. Each isotherm shows the heat of reaction, *Q* (circles), measured during titration of 35 mM n-octyl-β-D-glucopyranoside (OG) with 50 mM *E. coli* polar lipid extract. The saturation (SAT) and solubilization (SOL) boundaries are indicated by blue and red lines, respectively. In (**b**), the uncertainty in the SAT boundary due to the presence of KcsA is represented by a light blue shaded band. Inset: Representative thermogram showing differential power, Δ*p*, as a function of time (*t*). The discontinuity at approximately *t* = 10 h corresponds to an increase in the injection volume. Reproduced from [128] CC-BY.

To the best of our knowledge, no direct ITC studies on nanoghosts have been reported to date. However, several related investigations involving cell membranes derived from cells, particularly membrane reconstitution, offer valuable insights into the development of nanoghost-based drug delivery systems. For example, ITC was employed to monitor cell membrane–protein reconstitution in real time by titrating the potassium channel protein KcsA (tetramer) solubilized in n-octyl-β-D-glucopyranoside (OG), a non-ionic surfactant, into lipid extract from *E. coli* [120]. Figure 8 shows the ITC reconstitution isotherms of membrane lipid extracts with and without the KcsA tetramers. This work demonstrated the feasibility of using ITC to understand the impact of membrane proteins, such as KcsA, on the self-assembly of lipid bilayers in the presence of nonionic detergents [120]. Even at low concentrations, KcsA significantly influenced the supramolecular organization of the lipid–surfactant system, shifting the critical lipid-to-surfactant ratios for both the onset and completion of vesicle formation by more than twofold. the study demonstrated that ITC can be effectively used to monitor membrane–protein reconstitution under nonequilibrium conditions. This approach allows for real-time, noninvasive, and high-resolution tracking of the reconstitution process, ultimately enabling the formation of functional protein within liposomes.

ITC was employed to investigate the interactions between hydrolysable tannins and lipid vesicles derived from a phospholipid extract of *E. coli* [129]. In this study, a diverse set of 24 structurally distinct hydrolysable tannins was selected to identify structural features influencing their affinity for lipid vesicles in aqueous buffered media. In general, the interactions between hydrolysable tannins and lipid vesicles were exothermic in nature, and ITC can effectively screen hydrolysable tannins based on their lipid-binding affinities. Key structural determinants contributing to stronger lipid interactions included the presence of galloyl groups, increased structural flexibility, higher hydrophobicity, and greater molecular weight. Among the tested compounds, rugosins D and G exhibited the strongest interactions with the lipid vesicles [129]. Interestingly, certain tannins with moderate hydrophobicity, such as geraniin, chebulagic acid, and chebulinic acid, showed no detectable binding, suggesting that hydrophobicity alone is not sufficient to confer lipid affinity. These findings highlight the importance of multiple structural factors in governing tannin–lipid interactions.

### 3.8. ITC Applications in Native Cell Membrane and Live Cells

While ITC is widely used to investigate molecular interactions of biomimetic nanocarriers, its application has also been explored for live, whole cells, offering new insights into cellular processes in real time. Most available ITC studies involving live cells have been conducted using a single-injection model, in which heat production is measured following a single reactant injection. For example, using Chinese hamster ovary (CHO) cells overexpressing group I metabotropic glutamate receptors (mGluR1), an ITC study was performed to investigate the inhibitory effects of deuterium oxide (D_2_O) on the IP_3_-mediated Ca^2+^ release [130]. To isolate the heat flux specifically associated with mGluR1 activation, heat production measured from wild-type CHO cells (lacking mGluR1 expression) was used as a baseline and subtracted from the heat signal obtained from mGluR1-expressing CHO cells. Methodological note: When applying ITC to live cells, it is critical to account for substantial background heat signals such as metabolic heat. This intrinsic heat can obscure the heat changes arising from specific binding or signaling events. To minimize such effects, live cell ITC experiments are typically performed in either water or buffer, instead of cell growth medium, limiting cell growth and activities. Additionally, control titrations, such as buffer injections into cell suspensions or ligand injection into cells lacking receptor of interest, are performed to sever as background thermal powers [120,121,122]. For polydisperse vesicle samples, careful solvent matching between reference and sample cells and extended equilibration times help to minimize baseline noise. Without these precautions, spurious heat changes from metabolism, stirring, or dilution effects may mask the true interaction signal. By incorporating control comparisons and baseline subtraction, ITC can reliably probe complex systems like EVs and live cells, though the technique must be applied with particular attention to these interference factors [123,124]. D_2_O is known to influence protein activity, stability, and ligand binding through hydrogen–deuterium (H/D) exchange. Upon stimulation with 3,5-dihydroxyphenylglycine (DHPG), an mGluR1 agonist, a significant reduction in heat production was observed in mGluR1-expressing CHO cells in D_2_O compared to those in H_2_O (*p* < 0.01, Student’s *t*-test). This reduction in heat output is believed to be linked to diminished mGluR1-mediated Ca^2+^ release, resulting from D/H exchange [130]. This study not only demonstrated the feasibility of using ITC to monitor live cell signaling events but also highlighted the utility of deuteration as a tool to explore the role of protons in cellular signaling pathways [130].

**Figure 9:**
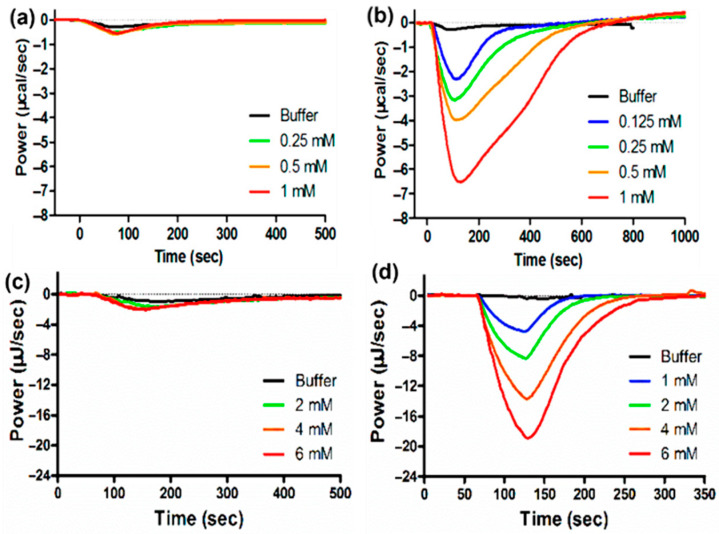
Overlaid heat flow curves comparing (**a**) cefazolin hydrolysis by *E. coli* cells lacking NDM-1 versus (**b**) *E. coli* cells expressing NDM-1, and (**c**) D-Ala-D-Ala hydrolysis by *E. coli* BL21 control cells versus (**d**) *E. coli* BL21 cells expressing VanX. (**a**,**b**) Reproduced with permission from [131], Copyright © 2018 American Chemical Society, (**c**,**d**) Reproduced with permission from [132], Elsevier, Copyright 2019.

Similarly, ITC has been successfully applied to monitor the enzymatic activity in live bacterial cells in real time. For instance, ITC was used to assess the activity of New Delhi metallo-β-lactamase 1 (NDM-1) expressed in live *E. coli* cells [131]. In this study, the hydrolysis of the β-lactam antibiotic cefazolin by NDM-1-expressing *E. coli* was monitored, while *E. coli* cells lacking NDM-1 served as controls. The heat released during the hydrolysis reaction was measured by ITC and directly correlated with the enzymatic activity of NDM-1 (Figure 9a,b). In addition to activity monitoring, the study evaluated the effectiveness of four known NDM-1 inhibitors—ethylene diamine tetraacetic acid (EDTA), D-captopril, ebselen, and azolylthioacetamide. The inhibitory effects were quantitatively assessed using ITC, and the resulting half-maximal inhibitory concentration values were found to be consistent with those obtained through conventional assays [131]. A similar ITC study was performed to effectively monitor the enzymatic activity of a D-alanyl-D-alanine dipeptidase, VanX, expressed on *E coli (*Figure 9c,d) [132]. These studies demonstrated the potential of cell-based ITC approach to screen and evaluate small-molecule inhibitors of bacterial enzymes and to potentially identify antibiotic-resistant bacterial strains.

Beyond enzymatic activity, ITC has also been employed to detect heat changes associated with the metabolic activity of cells [133]. Calorimetric thermograms have shown distinguishable features that can be used for rapid, qualitative, or semi-quantitative evaluation of growth-promoting or growth-inhibiting factors in defined microbial cultures. Additionally, thermal signatures can help identify different microbial growth phases. Overall, ITC has emerged as a promising analytical technique for the detection and investigation of antimicrobial resistance [134]. It has also been used for quantitative analysis of cell replication [135], assessment of release energy of various cancer cells [136], serving as an indirect measure of cellular metabolism and growth.

## 4. Conclusions

In summary, ITC has proven to be a powerful and effective tool for quantitatively studying a wide range of molecular interactions involving biomimetic nanocarriers. For solid lipid nanoparticles, ITC plays a crucial role of characterizing molecular interactions within formulations and with biological systems, such as drug–lipid matrix interactions and protein adsorption. Similarly, ITC studies of liposomes yield quantitative and mechanistic insights into drug–lipid interactions that are vital for optimizing drug delivery systems. ITC also facilitates formulation optimization by providing valuable information about membrane integrity and interactions with various drugs (e.g., peptides, therapeutic agents). Beyond its established applications in liposomes and solid lipid nanoparticles, this review aims to highlight and encourage further exploration of ITC in the study of EVs, cell membrane coated nanoparticles (nanoghosts). Its continued development could significantly impact on the future of EV-based diagnostics and nanoghosts-mediated drug delivery platforms. Furthermore, ITC has also demonstrated its applicability in studying live cells. Despite all potential benefits of ITC, it advisory to further validate the data with orthogonal techniques (e.g., spectroscopy, mass spectrometry, structural biology) when possible.

## Data Availability

No original research data, software or code have been included, and no new results were generated or analyzed as part of this review.

## Abbreviations

The following abbreviations are used in this manuscript:

ITC	Isothermal Titration Calorimetry
EVs	Extracellular Vesicles
RNA	Ribonucleic Acid
mRNA	Messenger RNA
BSA	Bovine Serum Albumin
HAS	Human Serum Albumin
DOPE	Dioleoylphosphatidylethanolamine
Dlin-MC3-DMA	Heptatriaconta-6,9,28,31-tetraen-19-yl 4-(dimethylamino)butanoate
DOPC	1,2-dioleoyl-sn-glycero-3-phosphocholine
DSPE-PEG2000	1,2-distearoyl-sn-glycero-3-phosphoethanolamine-poly(ethylene glycol)-2000
PLGA	Poly(lactic-co-glycolic acid)
ApoA1	Apolipoprotein A1
PC	Phosphatidylcholine
PE	Phosphatidylethanolamine
PA	Phosphatidic Acid
PS	Phosphatidylserine
POPC	1-palmitoyl-2-oleoyl-sn-glycero-3-phosphocholine
POPG	1-palmitoyl-2-oleoyl-sn-glycero-3-phospho-(1’-rac-glycerol)
DMPC	1,2-dimyristoyl-sn-glycero-3-phosphocholine
CL	Cardiolipin
DOTAP	1,2-dioleoyl-3-trimethylammoniumpropane
ANS	1-anilino-8-naphthalenesulfonate
TPB	Tetraphenylborate
DPPC	1,2-dipalmitoyl-sn-glycero-3-phosphocholine
DSPC	1,2-distearoyl-sn-glycero-3-phosphocholine
CPPs	Cell-Penetrating Peptides
TAT	Trans-Activator of Transcription
HIV-1	Human Immunodeficiency Virus type 1
NaCl	Sodium Chloride
Ca^2+^	Calcium ion
CHO	Chinese Hamster Ovary cells
mGluR1	Group I Metabotropic Glutamate Receptor 1
D_2_O	Deuterium Oxide
H_2_O	Water
D/H	Deuterium/Hydrogen exchange
DHPG	3,5-Dihydroxyphenylglycine
NDM-1	New Delhi Metallo-β-lactamase 1
EDTA	Ethylenediaminetetraacetic acid
VanX	D-alanyl-D-alanine dipeptidase
Aβ	Amyloid beta peptide

## References

[1] Sushnitha M., Evangelopoulos M., Tasciotti E., Taraballi F. (2020). Cell membrane-based biomimetic nanoparticles and the immune system: Immunomodulatory interactions to therapeutic applications. Front. Bioeng. Biotechnol..

[2] Motallebi M., Heidarizadeh F. (2024). Introduction to biomimetic nanoparticles for biomedical applications. Cell Membrane Surface-Engineered Nanoparticles: Biomimetic Nanomaterials for Biomedical Applications.

[3] Issaka E., Tornyeava B., Agyekum E.A., Enyan M., Okai Amu-Darko J.N., Chimuza H.T. (2024). Nature-inspired solutions: A comprehensive review of biomimetic nanoparticles in nanomedicine. J. Thermoplast. Compos. Mater..

[4] Vatankhah A., Oroojalian F., Hoseinzadeh Moghaddam S., Kesharwani P., Sahebkar A. (2025). State-of-the-art review on liposomes as versatile cancer vaccine delivery systems. J. Drug Deliv. Sci. Technol..

[5] Chen L., Hong W., Ren W., Xu T., Qian Z., He Z. (2021). Recent progress in targeted delivery vectors based on biomimetic nanoparticles. Signal Transduct. Target. Ther..

[6] Li H., Jin K., Luo M., Wang X., Zhu X., Liu X., Jiang T., Zhang Q., Wang S., Pang Z. (2019). Size dependency of circulation and biodistribution of biomimetic nanoparticles: Red blood cell membrane-coated nanoparticles. Cells.

[7] Gao W., Yang X., Lin Z., Gao S., He B., Mei D., Wang D., Yuan L., Zhang H., Dai W. (2016). The use of a hydrophobic binding peptide modified lipid nanocarrier improving tumor distribution and antitumor efficacy. J. Biomed. Nanotechnol..

[8] Zhang J., Liu B., Chen H., Zhang L., Jiang X. (2024). Application and method of surface plasmon resonance technology in the preparation and characterization of biomedical nanoparticle materials. Int. J. Nanomed..

[9] Behan J.A., Xie Z., Wang Y.F., Yang X., Aastrup T., Yan Y., Adumeau L., Dawson K.A. (2023). Quartz crystal microbalance method to measure nanoparticle-receptor interactions and evaluate nanoparticle design efficiency. JACS Au.

[10] Petty R.M., Ceresa L., Alexander E., Pham D., Sabnis N., Fudala R., Lacko A.G., Krishnamoorthy R.R., Gryczynski Z., Gryczynski I. (2025). Fluorescence resonance energy transfer for drug loading assessment in reconstituted high-density lipoprotein manoparticles. Int. J. Mol. Sci..

[11] Nowak J.S., Czarna A., Grudnik P., Grygier P., Pustelny K., Langer A., Dubin G. (2024). Microscale thermophoresis (MST) and spectral shift (SpS) in drug discovery. TrAC Trends Anal. Chem..

[12] Winiewska-Szajewska M., Poznański J. (2025). Differential scanning fluorimetry followed by microscale thermophoresis and/or isothermal titration calorimetry as an efficient tool for ligand screening. Biophys. Rev..

[13] Zhang C.H., Rodriguez E., Bi C., Zheng X.W., Suresh D., Suh K., Li Z., Elsebaei F., Hage D.S. (2018). High performance affinity chromatography and related separation methods for the analysis of biological and pharmaceutical agents. Analyst.

[14] Ostergaard J., Heegaard N.H.H. (2003). Capillary electrophoresis frontal analysis: Principles and applications for the study of drug-plasma protein binding. Electrophoresis.

[15] ÉEcija-Arenas A., Román-Pizarro V., Fernández-Romero J.M. (2021). Separation and characterization of liposomes using asymmetric flow field-flow fractionation with online multi-angle light scattering detection. J. Chromatogr. A.

[16] Menéndez M. (2020). Isothermal Titration Calorimetry: Principles and Applications. eLS.

[17] Su H., Xu Y. (2018). Application of ITC-Based characterization of thermodynamic and kinetic association of ligands with proteins in drug design. Front. Pharmacol..

[18] Chang J.W., Armaou A., Rioux R.M. (2021). Continuous injection isothermal titration calorimetry for in situ evaluation of thermodynamic binding properties of ligand–receptor binding models. J. Phys. Chem. B.

[19] Abian O., Vega S., Velazquez-Campoy A. (2023). Biological calorimetry: Old friend, new insights. Biophysica.

[20] Archer W.R., Schulz M.D. (2020). Isothermal titration calorimetry: Practical approaches and current applications in soft matter. Soft Matter.

[21] Lima Cavalcanti I.D., Xavier Junior F.H., Santos Magalhães N.S., Lira Nogueira M.C.B. (2023). Isothermal titration calorimetry (ITC) as a promising tool in pharmaceutical nanotechnology. Int. J. Pharm..

[22] Wang Y., Wang G., Moitessier N., Mittermaier A.K. (2020). Enzyme kinetics by isothermal titration calorimetry: Allostery, inhibition, and dynamics. Front. Mol. Biosci..

[23] Hagedoorn P.-L. (2022). Isothermal titration calorimetry in biocatalysis. Front. Catal..

[24] Prozeller D., Morsbach S., Landfester K. (2019). Isothermal titration calorimetry as a complementary method for investigating nanoparticle–protein interactions. Nanoscale.

[25] Swaine T.S., Garcia P., Tang Y., Lewis A.L., Parkes G., Waters L.J. (2019). Characterizing drug-polymer bead interactions using isothermal titration calorimetry. J. Pharm. Sci..

[26] Wieprecht T., Seelig J. (2002). Isothermal titration calorimetry for studying interactions between peptides and lipid membranes. Curr. Top. Membr..

[27] Rajarathnam K., Rösgen J. (2014). Isothermal titration calorimetry of membrane proteins—Progress and challenges. Biochim. Biophys. Acta (BBA)—Biomembr..

[28] Swamy M.J., Sankhala R.S., Singh B.P., Kleinschmidt J.H. (2019). Thermodynamic Analysis of Protein–Lipid Interactions by Isothermal Titration Calorimetry. Lipid-Protein Interactions: Methods and Protocols.

[29] Duff M.R., Grubbs J., Howell E.E. (2011). Isothermal titration calorimetry for measuring macromolecule-ligand affinity. J. Vis. Exp..

[30] Dunitz J.D. (1995). Win some, lose some: Enthalpy-entropy compensation in weak intermolecular interactions. Chem. Biol..

[31] Bronowska A.K., Moreno Piraján J.C. (2011). Thermodynamics of Ligand-Protein Interactions: Implications for Molecular Design. Thermodynamics—Interaction Studies—Solids, Liquids and Gases.

[32] Bouchemal K. (2008). New challenges for pharmaceutical formulations and drug delivery systems characterization using isothermal titration calorimetry. Drug Discov. Today.

[33] Tellinghuisen J. (2005). Optimizing experimental parameters in isothermal titration calorimetry. J. Phys. Chem. B.

[34] Sprakel L.M.J., Schuur B. (2019). Improving understanding of solvent effects on intermolecular interactions in reactive liquid–liquid extraction with Isothermal Titration Calorimetry and molecular modeling. J. Ind. Eng. Chem..

[35] Wangsakan A., Chinachoti P., McClements D.J. (2006). Isothermal titration calorimetry study of the influence of temperature, pH and salt on maltodextrin–anionic surfactant interactions. Food Hydrocoll..

[36] Mpye K.L., Gildenhuys S., Mosebi S. (2020). The effects of temperature on streptavidin-biotin binding using affinity isothermal titration calorimetry. AIMS Biophys..

[37] Erlekam F., Zumbansen M., Weber M. (2022). Parameter estimation on multivalent ITC data sets. Sci. Rep..

[38] Medoš Ž., Bešter-Rogač M., Leontidis E., Tellinghuisen J. (2024). Calibrating ITC instruments: Problems with weak base neutralization. Anal. Biochem..

[39] Le V.H., Buscaglia R., Chaires J.B., Lewis E.A. (2013). Modeling complex equilibria in isothermal titration calorimetry experiments: Thermodynamic parameters estimation for a three-binding-site model. Anal. Biochem..

[40] Brautigam C.A. (2015). Fitting two- and three-site binding models to isothermal titration calorimetric data. Methods.

[41] Chen J., Tian W., Yun Y., Tian Y., Sun C., Ding R., Chen H. (2021). A discussion on the affecting factors of the fitting procedures’ reliability in isothermal titration calorimetry analysis. Arch. Biochem. Biophys..

[42] Kantonen S.A., Henriksen N.M., Gilson M.K. (2017). Evaluation and minimization of uncertainty in itc binding measurements: Heat error, concentration error, saturation, and stoichiometry. Biochim. Biophys. Acta Gen. Subj..

[43] Baranauskienė L., Petrikaitė V., Matulienė J., Matulis D. (2009). Titration calorimetry standards and the precision of isothermal titration calorimetry data. Int. J. Mol. Sci..

[44] Wang T.Y., Bijlani A., Chao E.H.P., Johnson P.E., Krylov S.N. (2025). A browser-based tool for assessing accuracy of isothermal titration calorimetry-derived parameters: K(d), ΔH°, and n. Chembiochem.

[45] Leavitt S., Freire E. (2001). Direct measurement of protein binding energetics by isothermal titration calorimetry. Curr. Opin. Struct. Biol..

[46] Freyer M.W., Lewis E.A. (2008). Isothermal titration calorimetry: Experimental design, data analysis, and probing macromolecule/ligand binding and kinetic interactions. Methods Cell Biol..

[47] Velazquez-Campoy A., Freire E. (2006). Isothermal titration calorimetry to determine association constants for high-affinity ligands. Nat. Protoc..

[48] Duan Y., Dhar A., Patel C., Khimani M., Neogi S., Sharma P., Siva Kumar N., Vekariya R.L. (2020). A brief review on solid lipid nanoparticles: Part and parcel of contemporary drug delivery systems. RSC Adv..

[49] Swetha K., Kotla N.G., Tunki L., Jayaraj A., Bhargava S.K., Hu H., Bonam S.R., Kurapati R. (2023). Recent advances in the lipid nanoparticle-mediated delivery of mRNA vaccines. Vaccines.

[50] Schoenmaker L., Witzigmann D., Kulkarni J.A., Verbeke R., Kersten G., Jiskoot W., Crommelin D.J.A. (2021). mRNA-lipid nanoparticle COVID-19 vaccines: Structure and stability. Int. J. Pharm..

[51] Huang Z., Ma C., Wu M., Li X., Lu C., Zhang X., Ma X., Yang Y., Huang Y., Pan X. (2019). Exploring the drug-lipid interaction of weak-hydrophobic drug loaded solid lipid nanoparticles by isothermal titration calorimetry. J. Nanoparticle Res..

[52] Brader M.L., Williams S.J., Banks J.M., Hui W.H., Zhou Z.H., Jin L. (2021). Encapsulation state of messenger RNA inside lipid nanoparticles. Biophys. J..

[53] Pink D.L., Loruthai O., Ziolek R.M., Wasutrasawat P., Terry A.E., Lawrence M.J., Lorenz C.D. (2019). On the structure of solid lipid nanoparticles. Small.

[54] Rathee J., Kishore N. (2025). Interaction of solid lipid nanoparticles with bovine serum albumin: Physicochemical mechanistic insights. Phys. Chem. Chem. Phys..

[55] Miao L., Lin J., Huang Y., Li L., Delcassian D., Ge Y., Shi Y., Anderson D.G. (2020). Synergistic lipid compositions for albumin receptor mediated delivery of mRNA to the liver. Nat. Commun..

[56] Wang W., Huang Z., Li Y., Shi J., Fu F., Huang Y., Pan X., Wu C. (2021). Impact of particle size and pH on protein corona formation of solid lipid nanoparticles: A proof-of-concept study. Acta Pharm. Sin. B.

[57] Frank P.G., Marcel Y.L. (2000). Apolipoprotein A-I: Structure-function relationships. J. Lipid Res..

[58] Li M., Jin X., Liu T., Fan F., Gao F., Chai S., Yang L. (2022). Nanoparticle elasticity affects systemic circulation lifetime by modulating adsorption of apolipoprotein A-I in corona formation. Nat. Commun..

[59] Sawant R.R., Torchilin V.P. (2010). Liposomes as ‘smart’ pharmaceutical nanocarriers. Soft Matter.

[60] Nsairat H., Khater D., Sayed U., Odeh F., Al Bawab A., Alshaer W. (2022). Liposomes: Structure, composition, types, and clinical applications. Heliyon.

[61] Li S., Wang F., Li X., Chen J., Zhang X., Wang Y., Liu J. (2017). Dipole orientation matters: Longer-circulating choline phosphate than phosphocholine liposomes for enhanced tumor targeting. Acs Appl. Mater. Interfaces.

[62] Rampado R., Giordano F., Moracci L., Crotti S., Caliceti P., Agostini M., Taraballi F. (2022). Optimization of a detergent-based protocol for membrane proteins purification from mammalian cells. J. Pharm. Biomed. Anal..

[63] Sideratou Z., Tsiourvas D., Paleos C., Tsortos A., Nounesis G. (2000). Molecular recognition of complementary liposomes: The enhancing role of cholesterol. Langmuir.

[64] Caritá A., Cavalcanti R., Oliveira M., Riske K. (2023). Solubilization of biomimetic lipid mixtures by some commonly used non-ionic detergents. Chem. Phys. Lipids.

[65] Angerer N., Piller P., Semeraro E.F., Keller S., Pabst G. (2023). Interaction of detergent with complex mimics of bacterial membranes. Biophys. Chem..

[66] Krylova O.O., Jahnke N., Keller S. (2010). Membrane solubilisation and reconstitution by octylglucoside: Comparison of synthetic lipid and natural lipid extract by isothermal titration calorimetry. Biophys. Chem..

[67] Heerklotz H., Seelig J. (2000). Titration calorimetry of surfactant–membrane partitioning and membrane solubilization. Biochim. Biophys. Acta (BBA)—Biomembr..

[68] Heerklotz H., Lantzsch G., Binder H., Klose G., Blume A. (1995). Application of isothermal titration calorimetry for detecting lipid membrane solubilization. Chem. Phys. Lett..

[69] Heerklotz H., Tsamaloukas A.D., Keller S. (2009). Monitoring detergent-mediated solubilization and reconstitution of lipid membranes by isothermal titration calorimetry. Nat. Protoc..

[70] Clark S.T., Arras M.M.L., Sarles S.A., Frymier P.D. (2020). Lipid shape determination of detergent solubilization in mixed-lipid liposomes. Colloids Surf. B Biointerfaces.

[71] Yokoyama H., Ikeda K., Wakabayashi M., Ishihama Y., Nakano M. (2013). Effects of lipid membrane curvature on lipid packing state evaluated by isothermal titration calorimetry. Langmuir.

[72] Keller S., Heerklotz H., Jahnke N., Blume A. (2006). Thermodynamics of Lipid Membrane Solubilization by Sodium Dodecyl Sulfate. Biophys. J..

[73] Lombardo D., Kiselev M.A. (2022). Methods of liposomes preparation: Formation and control factors of versatile nanocarriers for biomedical and nanomedicine application. Pharmaceutics.

[74] Liu D., Chen W., Tasi L., Yang S. (2000). Microcalorimetric and shear studies on the effects of cholesterol on the physical stability of lipid vesicles. Colloids Surf. A Physicochem. Eng. Asp..

[75] Wu G., Lee K.Y.C. (2009). Interaction of poloxamers with liposomes: An isothermal titration calorimetry study. J. Phys. Chem. B.

[76] Solis-Gonzalez O.A., Ramon A.-G.J., Rojas-Aguilar A. (2022). A thermodynamic study of F108 and F127 block copolymer interactions with liposomes at physiological temperature. J. Liposome Res..

[77] Rai R., Kumar D., Dhule A.A., Rudani B.A., Tiwari S. (2024). Alkanols regulate the fluidity of phospholipid bilayer in accordance to their concentration and polarity. Langmuir.

[78] Šturm L., Poklar Ulrih N. (2021). Basic methods for preparation of liposomes and studying their interactions with different compounds, with the emphasis on polyphenols. Int. J. Mol. Sci..

[79] Roy B., Guha P., Nahak P., Karmakar G., Maity S., Mandal A., Bykov A.G., Akentiev A., Noskov B., Tsuchiya K. (2018). Biophysical correlates on the composition, functionality, and structure of dendrimer−liposome aggregates. ACS Omega.

[80] Mertins O., Dimova R. (2011). Binding of chitosan to phospholipid vesicles studied with isothermal titration calorimetry. Langmuir.

[81] Navon Y., Radavidson H., Putaux J.-L., Jean B., Heux L. (2017). pH-sensitive interactions between cellulose nanocrystals and DOPC liposomes. Biomacromolecules.

[82] Samelo J., Mora M.J., Granero G.E., Moreno M.J. (2017). Partition of amphiphilic molecules to lipid bilayers by ITC: Low-affinity solutes. ACS Omega.

[83] Vargas C., Klingler J., Keller S., Rapaport D., Herrmann J.M. (2013). Membrane Partitioning and Translocation Studied by Isothermal Titration Calorimetry. Membrane Biogenesis: Methods and Protocols.

[84] Tsamaloukas A.D., Keller S., Heerklotz H. (2007). Uptake and release protocol for assessing membrane binding and permeation by way of isothermal titration calorimetry. Nat. Protoc..

[85] Osanai H., Ikehara T., Miyauchi S., Shimono K., Tamogami J., Nara T., Kamo N. (2013). A study of the interaction of drugs with liposomes with isothermal titration calorimetry. J. Biophys. Chem..

[86] Kaur H., Kishore N. (2025). Anti-cancer and anti-microbial drug encapsulated lipid vesicles as drug delivery systems: Calorimetric and spectroscopic study. Colloids Surf. A Physicochem. Eng. Asp..

[87] Matos C., Lima J.L., Reis S., Lopes A., Bastos M. (2004). Interaction of antiinflammatory drugs with EPC liposomes: Calorimetric study in a broad concentration range. Biophys. J..

[88] Nordstrom R., Zhu L., Harmark J., Levi-Kalisman Y., Koren E., Barenholz Y., Levinton G., Shamrakov D. (2021). Quantitative cryo-TEM reveals new structural details of Doxil-like pegylated liposomal doxorubicin formulation. Pharmaceutics.

[89] Ben-Fadhel Y., Maherani B., Salmieri S., Lacroix M. (2022). Preparation and characterization of natural extracts-loaded food grade nanoliposomes. LWT.

[90] Zhang M., Meng J.-Y., Wang Y.-N., Wu R.-G. (2025). Investigation on the interaction between Jingui Shenqi pill and DPPC liposome mimetic biofilms. J. Therm. Anal. Calorim..

[91] Cordeiro M., Filipe H., dos Santos P., Samelo J., Ramalho J., Loura L., Moreno M. (2023). Interaction of Hoechst 33342 with POPC membranes at different pH values. Molecules.

[92] Boruah J.S., Chowdhury D. (2022). Liposome-azobenzene nanocomposite as photo-responsive drug delivery vehicle. Appl. Nanosci..

[93] Has C., Das S.L. (2023). The functionality of membrane-inserting proteins and peptides: Curvature sensing, generation, and pore formation. J. Membr. Biol..

[94] Kim S., Lee J., Lee S., Kim H., Sim J.-Y., Pak B., Kim K., Kim J.I. (2022). Matching amino acids membrane preference profile to improve activity of antimicrobial peptides. Commun. Biol..

[95] Henriksen J.R., Andresen T.L. (2011). Thermodynamic profiling of peptide membrane interactions by isothermal titration calorimetry: A search for pores and micelles. Biophys. J..

[96] Kumagai P.S., Sousa V.K., Donato M., Itri R., Beltramini L.M., Araujo A.P.U., Buerck J., Wallace B.A., Lopes J.L.S. (2019). Unveiling the binding and orientation of the antimicrobial peptide Plantaricin 149 in zwitterionic and negatively charged membranes. Eur. Biophys. J..

[97] Vieira A.P., de Souza A.N., Lima W.G., Brito J.C., Simião D.C., Gonçalves L.V., Cordeiro L.P., de Oliveira Scoaris D., Fernandes S.O., Resende J.M. (2024). The synthetic peptide LyeTx I mn∆K, derived from Lycosa erythrognatha spider toxin, is active against methicillin-resistant staphylococcus aureus (MRSA) in vitro and in vivo. Antibiotics.

[98] Beck K., Nandy J., Hoernke M. (2023). Membrane permeabilization can be crucially biased by a fusogenic lipid composition—Leaky fusion caused by antimicrobial peptides in model membranes. Soft Matter.

[99] Stulz A., Breitsamer M., Winter G., Heerklotz H. (2020). Primary and secondary binding of exenatide to liposomes. Biophys. J..

[100] Valkova I., Todorov P., Vitkova V. (2022). VV-hemorphin-5 association to lipid bilayers and alterations of membrane bending rigidity. Aims Biophys..

[101] Sonju J.J., Dahal A., Singh S.S., Jois S.D. (2021). Peptide-functionalized liposomes as therapeutic and diagnostic tools for cancer treatment. J. Control Release.

[102] Sauder R., Seelig J., Ziegler A., Langel Ü. (2011). Thermodynamics of Lipid Interactions with Cell-Penetrating Peptides. Cell-Penetrating Peptides: Methods and Protocols.

[103] Ziegler A., Li Blatter X., Seelig A., Seelig J. (2003). Protein transduction domains of HIV-1 and SIV TAT interact with charged lipid vesicles: Binding mechanism and thermodynamic analysis. Biochemistry.

[104] Takechi Y., Tanaka H., Kitayama H., Yoshii H., Tanaka M., Saito H. (2012). Comparative study on the interaction of cell-penetrating polycationic polymers with lipid membranes. Chem. Phys. Lipids.

[105] Cavalcanti R., Lira R., Riske K. (2022). Membrane fusion biophysical analysis of fusogenic liposomes. Langmuir.

[106] Wu S., Jiang P., Zhang X., Mao C., Dai Y., Zhuang H., Pang Y. (2024). Understanding the transepithelial transport and transbilayer diffusion of the antihypertensive peptide Asn-Cys-Trp: Insights from Caco-2 Cell monolayers and the DPPC model membrane. J. Agric. Food Chem..

[107] Terakawa M.S., Yagi H., Adachi M., Lee Y.-H., Goto Y. (2015). Small liposomes accelerate the fibrillation of amyloid β (1–40). J. Biol. Chem..

[108] Dimitrova M., Matsumura H., Terezova N., Neytchev V. (2002). Binding of globular proteins to lipid membranes studied by isothermal titration calorimetry and fluorescence. Colloids Surf. B-Biointerfaces.

[109] Anbazhagan V., Sankhala R.S., Singh B.P., Swamy M.J. (2011). Isothermal titration calorimetric studies on the interaction of the major bovine seminal plasma protein, PDC-109 with phospholipid membranes. PLoS ONE.

[110] Sangrà M., Estelrich J., Sabaté R., Espargaró A., Busquets M.A. (2017). Evidence of protein adsorption in pegylated liposomes: Influence of liposomal decoration. Nanomaterials.

[111] Nele V., D’Aria F., Campani V., Silvestri T., Biondi M., Giancola C., De Rosa G. (2024). Unravelling the role of lipid composition on liposome-protein interactions. J. Liposome Res..

[112] Arseneault M., Lafleur M. (2006). Isothermal titration calorimetric study of calcium association to lipid bilayers: Influence of the vesicle preparation and composition. Chem. Phys. Lipids.

[113] Miller D., Booth P.J. (2010). The use of isothermal titration calorimetry to study multidrug transport proteins in liposomes. Methods Mol. Biol..

[114] Boudker O., Oh S. (2015). Isothermal titration calorimetry of ion-coupled membrane transporters. Methods.

[115] Ha D., Yang N.N., Nadithe V. (2016). Exosomes as therapeutic drug carriers and delivery vehicles across biological membranes: Current perspectives and future challenges. Acta Pharm. Sin. B.

[116] Luan X., Sansanaphongpricha K., Myers I., Chen H.W., Yuan H.B., Sun D.X. (2017). Engineering exosomes as refined biological nanoplatforms for drug delivery. Acta Pharmacol. Sin..

[117] Horibe S., Tanahashi T., Kawauchi S., Murakami Y., Rikitake Y. (2018). Mechanism of recipient cell-dependent differences in exosome uptake. BMC Cancer.

[118] Narain A., Asawa S., Chhabria V., Patil-Sen Y. (2017). Cell membrane coated nanoparticles: Next-generation therapeutics. Nanomedicine.

[119] Gao W.W., Zhang L.F. (2015). Coating nanoparticles with cell membranes for targeted drug delivery. J. Drug Target..

[120] Hessvik N.P., Llorente A. (2018). Current knowledge on exosome biogenesis and release. Cell. Mol. Life Sci..

[121] Thery C., Zitvogel L., Amigorena S. (2002). Exosomes: Composition, biogenesis and function. Nat. Rev. Immunol..

[122] Hu C.M.J., Zhang L., Aryal S., Cheung C., Fang R.H., Zhang L.F. (2011). Erythrocyte membrane-camouflaged polymeric nanoparticles as a biomimetic delivery platform. Proc. Natl. Acad. Sci. USA.

[123] Meng Q.F., Rao L., Zan M.H., Chen M., Yu G.T., Wei X.Y., Wu Z.H., Sun Y., Guo S.S., Zhao X.Z. (2018). Macrophage membrane-coated iron oxide nanoparticles for enhanced photothermal tumor therapy. Nanotechnology.

[124] Elliott R.O., He M. (2021). Unlocking the power of exosomes for crossing biological barriers in drug delivery. Pharmaceutics.

[125] Li R.X., He Y.W., Zhang S.Y., Qin J., Wang J.X. (2018). Cell membrane-based nanoparticles: A new biomimetic platform for tumor diagnosis and treatment. Acta Pharm. Sin. B.

[126] Kroll A.V., Fang R.H., Zhang L.F. (2017). Biointerfacing and applications of cell membrane-coated nanoparticles. Bioconjugate Chem..

[127] Coughlan C., Lindenberger J., Jacot J.G., Johnson N.R., Anton P., Bevers S., Welty R., Graner M.W., Potter H. (2024). Specific binding of Alzheimer’s Aβ peptides to extracellular vesicles. Int. J. Mol. Sci..

[128] Jahnke N., Krylova O.O., Hoomann T., Vargas C., Fiedler S., Pohl P., Keller S. (2014). Real-time monitoring of membrane-protein reconstitution by isothermal titration calorimetry. Anal. Chem..

[129] Virtanen V., Green R.J., Karonen M. (2022). Karonen, M. Interactions between Hydrolysable tannins and lipid vesicles from *Escherichia coli* with isothermal titration calorimetry. Molecules.

[130] Hirakura Y., Sugiyama T., Takeda M., Ikeda M., Yoshioka T. (2011). Deuteration as a tool in investigating the role of protons in cell signaling. Biochim. Biophys. Acta (BBA)—Gen. Subj..

[131] Zhang Y.-J., Wang W.-M., Oelschlaeger P., Chen C., Lei J.-E., Lv M., Yang K.-W. (2018). Real-time monitoring of NDM-1 activity in live bacterial cells by isothermal titration calorimetry: A new approach to measure inhibition of antibiotic-resistant bacteria. ACS Infect. Dis..

[132] Lv M., Zhang Y.-J., Zhou F., Ge Y., Zhao M.-H., Liu Y., Yang K.-W. (2019). Real-time monitoring of D-Ala-D-Ala dipeptidase activity of VanX in living bacteria by isothermal titration calorimetry. Anal. Biochem..

[133] Matulis D., Wadsö L., Fahmy K. (2023). Special Issue “advances in monitoring metabolic activities of microorganisms by calorimetry”. Microorganisms.

[134] Patnaik A., Rai S.K., Dhaked R.K. (2025). Analytical techniques and molecular platforms for detection and surveillance of antimicrobial resistance: Advancements of the past decade. 3 Biotech.

[135] Fahmy K. (2022). Simple growth–metabolism relations are revealed by conserved patterns of heat flow from cultured microorganisms. Microorganisms.

[136] Lemos D., Oliveira T., Martins L., de Azevedo V.R., Rodrigues M.F., Ketzer L.A., Rumjanek F.D. (2020). Isothermal microcalorimetry of tumor cells: Enhanced thermogenesis by metastatic cells. Front. Oncol..

